# Supplementary material for: A bistable prokaryotic differentiation system underlying development of conjugative transfer competence
Source: PLoS Genet. 2022 Jun 28;18(6):e1010286. doi: 10.1371/journal.pgen.1010286 (PMC9286271; doi:10.1371/journal.pgen.1010286)
Supplement: S1 Table — (DOCX) [file pgen.1010286.s001.docx]

Supplementary table 1: Strain specifications.

| **Strain number** | **Description** | **Remarks** | **Source or Reference** |
| --- | --- | --- | --- |
| **78** | *P. knackmussii* B13 | Original host for ICE*clc* | ^1^ |
| **1291** | *P. putida* UWC1 | ICE*clc*-free control strain | ^2^ |
| **1343** | *P. knackmussii* B13 P_int_-egfp, Km^R^ | Single copy *intB13* promoter-egfp fusion insertion | ^3^ |
| **2058** | *P. knackmussii* B13 *inrR*^+/-^ | Deletion of one copy of *inrR* | ^4^ |
| **2201** | *P. knackmussii* B13 *inrR*^-/-^ | Deletion of both copies of *inrR* | ^4^ |
| **2397-98** | *P. knackmussii* B13 P_inR_-egfp, Km^R^ | Single copy *inrR* promoter-egfp fusion insertion | ^4^ |
| **2433** | *P. putida* UWC1 (ICE*clc-∆inrR*) | By conjugation from 2201 | ^4^ |
| **2580-82** | *P. knackmussii* B13 P_int_-egfp/P_inR_-*mcherry*, Km^R^ | Single copy bidirectional *intB13* promoter-egfp*/inrR* promoter-*mcherry* fusion insertion | ^4^ |
| **2737,2738** | *P. putida* UWC1 (ICE*clc*) | ICE*clc* element in 1291, tRNA^Gly^ | ^5^ |
| **2744** | *P. putida* UWC1, mini-Tn7-P_tac_-*mCherry*-Gm^R^ | Constitutive mCherry expression, used as mating recipient | ^5^ |
| **2748** | *P. knackmussii* B13, mini-Tn5(P_int_-mcherry) Tet^R^ | P_int_ reporter in 78  262 bp fragment | ^4^ |
| **3044** | *E. coli* DH5α λpir | Host for propagation plasmids with R6K origin of replication | Victor de Lorenzo |
| **3734** | *E. coli* DH5α λpir, mini-Tn5 P(*orf81655*)-egfp, Ap^R^ Km^R^ | pBAM-egfp containing promoter region of *orf81655* | This study |
| **3737** | *E. coli* DH5α λpir, mini-Tn5 P(*orf81655*)-egfp Km^R^ | pBAM-egfp containing promoter region of *orf81655* (fragment 4) | This study |
| **3741-3743** | *P. knackmussii* B13, mini-Tn5(P_81655_-egfp*)*, Km^R^ | Single copy insertion of (P_81655_-egfp*)* reporter of 3734 in 78 | This study |
| **3955** | *E. coli* DH5α λpir, mini-Tn5 P(*orf100952*)-egfp Km^R^ | pBAM-egfp containing promoter region of *orf100952 (alpA)* | This study |
| **3956-58** | *P. knackmussii* B13, mini-Tn5 P(*orf100952*)-egfp Km^R^ | P_alpA_-egfp reporter from 3955 in 78 | This study |
| **3960** | *E. coli* DH5α λpir, mini-Tn5 P(*orf67231*)-egfp Km^R^ | pBAM-egfp containing promoter region of *orf67231* | This study |
| **3961-63** | *P. knackmussii* B13, mini-Tn5 P(*orf67231*)-egfp Km^R^ | P*_orf67231-_*-egfp reporter from 3960 in 78 | This study |
| **3965** | *E. coli* DH5α λpir, mini-Tn5 P(*orf67800*)-egfp Km^R^ | pBAM-egfp containing promoter region of *orf67800* | This study |
| **3966-68** | *P. knackmussii* B13, mini-Tn5 P(*orf67800*)-egfp Km^R^ | P*_orf67800_*-egfp reporter from strain 3965 in 78 | This study |
| **3970** | *E. coli* DH5α λpir, mini-Tn5 UR(*orf89746*)-egfp Km^R^ | pBAM-egfp (strain 3726) containing upstream region of *orf89746* | This study |
| **3971-73** | *P. knackmussii* B13, mini-Tn5 UR(*orf89746*)-egfp Km^R^ | UR*_orf89746_*-egfp reporter from 3970 in strain 78 | This study |
| **3991** | *E. coli* DH5α λpir, mini-Tn5 UR(*orf66202*)-egfp Km^R^ | pBAM-egfp containing upstream region of *orf66202* | This study |
| **3992-94** | *P. knackmussii* B13, mini-Tn5 UR(*orf66202*)-egfp Km^R^ | *_URorf66202_*-egfp reporter from 3991 in 78 | This study |
| **3996** | *E. coli* DH5α λpir, mini-Tn5 UR(*traI*)-egfp Km^R^ | pBAM-egfp (strain 3726) containing short upstream region of *traI* | This study |
| **3997-99** | *P. knackmussii* B13, mini-Tn5 UR(*traI*)-egfp Km^R^ | *_URtraI_*-egfp reporter from 3996 in 78 | This study |
| **4001** | *E. coli* DH5α λpir, mini-Tn5 P(*orf88400*)-egfp Km^R^ | pBAM-egfp containing promoter region of *orf88400* | This study |
| **4002-04** | *P. knackmussii* B13, mini-Tn5 P(*orf88400*)-egfp Km^R^ | P*_orf88400_*-egfp reporter from 4001 in 78 | This study |
| **4006** | *E. coli* DH5α λpir, mini-Tn5 P(*orf101284*)-egfp Km^R^ | pBAM-egfp containing promoter region of *orf101284* (*bisR*) | ^6^ |
| **4007-09** | *P. knackmussii* B13, mini-Tn5 P(*orf101284*)-egfp Km^R^ | P_bisR_-egfp reporter from 4006 in 78 | This study |
| **4011** | *E. coli* DH5α λpir, mini-Tn5 UR(*orf100033*)-egfp Km^R^ | pBAM-egfp containing (short) upstream region of *orf100033* | This study |
| **4012-14** | *P. knackmussii* B13, mini-Tn5 UR(*orf100033*)-egfp Km^R^ | UR_100033_-egfp reporter from 4011 in 78 | This study |
| **4064** | *E. coli* DH5α λpir, mini-Tn5 UR(*orf84835*)-egfp Km^R^ | pBAM-egfp containing upstream region of *orf84835* | This study |
| **4065-67** | *P. knackmussii* B13, mini-Tn5 UR(*orf84835*)-egfp Km^R^ | *_URorf84835_*-egfp reporter of 4064 in 78 | This study |
| **4096-98** | *P. knackmussii* B13, mini-Tn5 P(*orf81655_4*)-egfp , P_int_-*eChe*, Tc,Km^R^ | *P_orf81655_*-egfp fragment–4 reporter of 3737 in 2748 | This study |
| **4122** | *E. coli* DH5α λpir, mini-Tn5 UR(*orf62755*)-egfp Km^R^ | pBAM-egfp containing upstream region of *orf62755* | This study |
| **4123-25** | *P. knackmussii* B13, mini-Tn5 UR(*orf62755*)-egfp Km^R^ | UR*_62755_*-egfp reporter of 4122 in 78 | This study |
| **4126-28** | *P. knackmussii* B13, mini-Tn5 P(*orf100952*)-egfp Km^R^, mini-Tn5(P_int_-mcherry) Tet^R^ | P*_orf100952_*-egfp reporter in 2748 (double reporter) | This study |
| **4129-31** | *P. knackmussii* B13, mini-Tn5 P(*orf67231*)-egfp Km^R^, mini-Tn5(P_int_-mcherry) Tet^R^ | P*_orf67231_*-egfp reporter in 2748 (double reporter) | This study |
| **4322** | *P. putida* UWC1 ICE*clc* ∆*mfsR* | Deletion of *mfsR*, used for RNAseq | ^7^ |
| **4323-25** | *P. knackmussii* B13, mini-Tn5 UR(*orf100033*)-egfp Km^R^, mini-Tn5(P_int_-mcherry) Tet^R^ | UR*_orf100033_*-egfp reporter in 2748 | This study |
| **4326-28** | *P. knackmussii* B13, mini-Tn5 P(*orf101284*)-egfp Km^R^, mini-Tn5(P_int_-mcherry) Tet^R^ | P*_orf101284_*-egfp reporter in 2748 (double reporter) | This study |
| **4369-71** | *P. knackmussii* B13, mini-Tn5 P(*orf88400*)-egfp Km^R^, mini-Tn5(P_int_-mcherry) Tet^R^ | P*_orf88400_*-egfp reporter in 2748 (double reporter) | This study |
| **4764-66** | *P. knackmussii* B13 *inrR*^+/-^, mini-Tn5 P(*orf88400*)-egfp Km^R^ | P*_orf88400_*-egfp reporter in strain 2058 | This study |
| **4767-69** | *P. knackmussii* B13 *inrR*^-/-^, mini-Tn5 P(*orf88400*)-egfp Km^R^ | P*_orf88400_*-egfp reporter in strain 2201 | This study |
| **4770-72** | *P. knackmussii* B13 *inrR*^+/-^, mini-Tn5 P(*orf100952*)-egfp Km^R^ | P*_orf100952_*-egfp reporter in strain 2058 | This study |
| **4773-75** | *P. knackmussii* B13 *inrR*^-/-^, mini-Tn5 P(*orf100952*)-egfp Km^R^ | P*_orf100952_*-egfp reporter in strain 2201 | This study |
| **4776-78** | *P. knackmussii* B13 *inrR*^+/-^, mini-Tn5 P(*orf101284*)-egfp Km^R^ | P*_orf101284_*-egfp reporter in strain 2058 | This study |
| **4779-81** | *P. knackmussii* B13 *inrR*^-/-^, mini-Tn5 P(*orf101284*)-egfp Km^R^ | P*_orf101284_*-egfp reporter in strain 2201 | This study |
| **4782-84** | *P. knackmussii* B13 *inrR*^+/-^, mini-Tn5 P(*orf81655*)-egfp Km^R^ | P*_orf81655_*-egfp reporter in strain 2058 | This study |
| **4785-87** | *P. knackmussii* B13 *inrR*^-/-^, mini-Tn5 P(*orf81655*)-egfp Km^R^ | P*_orf81655_*-egfp reporter in strain 2201 | This study |
| **4822-24** | *P. knackmussii* B13 *inrR*^+/-^, mini-Tn5 P(*orf67231*)-egfp Km^R^ | P*_orf67231_*-egfp reporter in strain 2058 | This study |
| **4826-28** | *P. knackmussii* B13 *inr*^-/-^, mini-Tn5 P(*orf67231*)-egfp Km^R^ | P*_orf67231_*-egfp reporter in strain 2201 | This study |
| **4852-54** | *P. putida* UWC1 *∆mfsR* P(*orf88400*)-egfp Km^R^ | P*_orf88400_*-egfp reporter in 4322 | This study |
| **4855-57** | *P. putida* UWC1 *∆mfsR* P(*orf100952*)-egfp Km^R^ | P*_orf100952_*-egfp reporter in 4322 | This study |
| **4858-60** | *P. putida* UWC1 *∆mfsR* P(*orf101284*)-egfp Km^R^ | P*_orf101284_*-egfp reporter in 4322 | This study |
| **4882-84** | *P. putida* UWC1 *∆mfsR* P(*orf81655*)-egfp Km^R^ | P*_orf81655_*-egfp reporter in 4322 | This study |
| **5284** | *E. coli* DH5a-lambda pir P(orf58432)-egfp, Ap^R^ | P*_orf58432_*-egfp reporter in pBAM | This study |
| **5294-96** | *P. putida* UWC1 *∆mfsR* P(*orf67231*)-egfp Km^R^ | P*_orf67231_*-egfp reporter in 4322 | This study |
| **5337-39** | *P. knackmussii* B13 mini-Tn5 P(*orf58432*)-egfp Km^R^ | P(*orf58432*)-egfp reporter from 5284 in 78 | This study |
| **5501** | *P. putida* UWC1 miniTn7::P(*int*)-egfp Gm^R^, pME6032 Tet^R^ | P*_int_*-egfp, empty plasmid pME6032 | ^6^ |
| **5502** | *P. putida* UWC1 miniTn7::P(*inR*)-egfp Gm^R^, pME6032 Tet^R^ | P*_inR_*-egfp, empty plasmid pME6032 | ^6^ |
| **5503** | *P. putida* UWC1 miniTn7::P(*alpA*)-egfp Gm^R^, pME6032 Tet^R^ | P*_alpA_*-egfp, empty plasmid pME6032 | ^6^ |
| **5553** | *P. putida* UWC1 clc5, ICE*clc* ∆*mfsR* ∆*bisR* | Derivative of 4322 with additional *bisR* deletion, used for RNAseq | This study |
| **5719, 5725, 5731** | *P. putida* UWC1 P_int_-*mcherry*/P_inR_-*egfp*, pME6032, Km^R^, Tc^R^ | Control for direction induction of Pint,PinR by BisDC | This study |
| **5929** | *E. coli* DH5α λpir, mini-Tn5 UR(*orf97571*)-egfp Km^R^ | pBAM-egfp containing upstream region of *orf97571* | This study |
| **5930-32** | *P. knackmussii* B13 mini-Tn5-UR(*orf97571*)-egfp insertion | UR(*orf97571)*-*egfp* reporter of 5929 in strain 78 | This study |
| **5933** | *E. coli* DH5α λpir, mini-Tn5 UR(*orf100033*)-egfp Km^R^ | pBAM-egfp containing upstream region of *orf100033* | This study |
| **5934-36** | *P. knackmussii* B13 mini-Tn5-UR(*orf100033*)-egfp insertion | UR(*orf100033)*-*egfp* reporter of 5933 in strain 78 | This study |
| **5937-39** | *P. knackmussii* B13 *inrR*^-/-^, mini-Tn5 P(inR)-egfp Km^R^ | PinR-egfp reporter from 2012 into 2201 | This study |
| **5940-42** | *P. knackmussii* B13 miniTn5-P_int_-*mcherry*; mini-Tn5-P(*orf58432*)-egfp insertion | P(*orf58432)*-*egfp* reporter of 5248 in strain 2748 | This study |
| **6059-61** | *P. putida* UWC1 P_int_-*mcherry*/P_inR_-*egfp*, pMEbisDC, Km^R^, Tc^R^ | Strain 5690 with plasmid from 6055 | This study |
| **6065** | *P. putida* UWC1 miniTn7::P(*alpA*)-egfp Gm^R^, pMEbisDC Tet^R^ | P*_alpA_*-egfp, bisDC expression from pMEbisDC | ^6^ |
| **6077** | *P. putida* UWC1 miniTn7::P(*int*)-egfp Gm^R^, pMEbisDC Tet^R^ | P*_int_*-egfp, bisDC expression from pMEbisDC | ^6^ |
| **6178-80** | *P. knackmussii* B13 *inrR*^+/-^, mini-Tn5 P(inR)-egfp Km^R^ | PinR-egfp reporter from 2012 into 2058 | This study |
| **6298-6300** | *P. putida* UWC1 miniTn5::P(*orf67231*)-egfp Km^R^, pME6032 Tet^R^ | P*_orf67231_*-egfp reporter in 1291, empty plasmid pME6032 | This study |
| **6301-03** | *P. putida* UWC1 miniTn5::P(*orf67231*)-egfp Km^R^, pMEbisDC Tet^R^ | P*_orf67231_*-egfp reporter in 1291, bisDC expression from pMEbisDC | This study |
| **6304-06** | *P. putida* UWC1 miniTn5::UR(*orf89746*)-egfp Km^R^, pME6032 Tet^R^ | UR*_orf89746_*-egfp reporter in 1291, empty plasmid pME6032 | This study |
| **6307-09** | *P. putida* UWC1 miniTn5::UR(*orf89746*)-egfp Km^R^, pMEbisDC Tet^R^ | UR*_orf89746_*-egfp reporter in 1291, bisDC expression from pMEbisDC | This study |
| **6310-12** | *P. putida* UWC1 miniTn5::P(*orf88400*)-egfp Km^R^, pME6032 Tet^R^ | P*_orf88400_*-egfp reporter in 1291, empty plasmid pME6032 | This study |
| **6313-15** | *P. putida* UWC1 miniTn5::P(*orf88400*)-egfp Km^R^, pMEbisDC Tet^R^ | P*_orf88400_*-egfp reporter in 1291, bisDC expression from pMEbisDC | This study |
| **6856** | *P. putida* UWC1 miniTn7::P(*inR*)-egfp Gm^R^, pMEbisDC Tet^R^ | P*_inR_*-egfp, bisDC expression from pMEbisDC | ^6^ |
| **7150** | *E. coli* DH5α λpir, *mini-Tn5 (PtraI)-egfp* Km^R^ | pBAM-egfp containing long promoter region of *traI* | This study |
| **7177-79** | *P. putida* UWC1 ICEclc5 miniTn5::P(*traI*)-egfp Km^R^ | P*_traI_*-egfp reporter of 7150 in 2737 | This study |
| **7181-82** | *P. putida* UWC1 miniTn5::P(*traI*)-egfp Km^R^, pMEbisDC Tet^R^ | P*_traI_*-egfp reporter (short version) of 7149 in 1291, bisDC expression from pMEbisDC | This study |
| **7183-84** | *P. putida* UWC1 miniTn5::P(*traI*)-egfp Km^R^, pMEbisDC Tet^R^ | P*_traI_*-egfp reporter of 7150 in 1291, bisDC expression from pMEbisDC | This study |
| **7205-06** | *P. putida* UWC1 miniTn5::P(*traI*)-egfp, Km^R^, pME6032 Tet^R^ | P*_traI_*-egfp reporter from 7150 in 1291, empty plasmid pME6032 | This study |
| **7310** | *E. coli* DH5α λpir, *mini-Tn5 (UR84835)-egfp* Km^R^ | pBAM-egfp (strain 3726) containing upstream region of *orf84835* | This study |
| **7311** | *E. coli* DH5α λpir, *mini-Tn5 (UR89247)-egfp* Km^R^ | pBAM-egfp (strain 3726) containing upstream region of *orf89247* | This study |
| **7330-32** | *P. putida* UWC1 ICEclc5 miniTn5::UR(*orf84835*)-egfp Km^R^ | *_URorf84835_*-egfp reporter from 7310 in 2737 | This study |
| **7334-36** | *P. putida* UWC1 ICEclc5 miniTn5::UR(*orf89247*)-egfp Km^R^ | *_URorf89247_*-egfp reporter from 7311 in 2737 | This study |
| **7378-80** | *P. putida* UWC1 ICEclc6 Δ81655-75419 mini-Tn5(PinR-egfp/Pint-mcherry) Km^R^ | P*_inR_*-egfp/ P*_int_*-mcherry reporter in 2738 carrying ICE*clc* deleted for orfs *81655-75419* | This study |
| **7381-83** | *P. putida* UWC1 ICEclc6 Δ88400-84388 mini-Tn5(PinR-egfp/Pint-mcherry) Km^R^ | P*_inR_*-egfp/ P*_int_*-mcherry reporter in 2738 carrying ICE*clc* deleted for orfs *88400-84388* | This study |
| **7384-86** | *P. putida* UWC1 ICEclc5 mini-Tn5(PinR-egfp/Pint-mcherry) Km^R^ | P*_inR_*-egfp/ P*_int_*-mcherry reporter in 2737 | This study |
| **7387-89** | *P. putida* UWC1 ICEclc6 Δ74436-68241 mini-Tn5(PinR-egfp/Pint-mcherry) Km^R^ | P*_inR_*-egfp/ P*_int_*-mcherry reporter in 2738 carrying ICE*clc* deleted for orfs *74436-68241* | This study |
| **7418** | *E. coli* DH5α λpir, mini-Tn5*::UR(73676)-egfp* Km^R^ | pBAM-egfp containing upstream region of *orf73676* | This study |
| **7423-25** | *P. putida* UWC1 ICEclc5 miniTn5::UR(*orf73676*)-egfp Km^R^ | UR*_orf73676_*-egfp reporter from 7418 in 2737 | This study |
| **7476-78** | *P. putida* UWC1 miniTn5::P(*orf81655 fgt4*)-egfp Km^R^, pME6032 Tet^R^ | P*_orf81655_*-egfp reporter from 3448 in 1291, empty plasmid pME6032 | This study |
| **7479-81** | *P. putida* UWC1 miniTn5::P(*orf81655 fgt4*)-egfp Km^R^ , pMEbisDC Tet^R^ | P*_orf81655_*-egfp reporter from 3448 in 1291, bisDC expression from pMEbisDC | This study |
| **7558-60** | *P. putida* UWC1 miniTn5::P(*traI*)-egfp, miniTn5::P_int_-*mcherry* Km^R^ , Tet^R^ | Double reporter fusion, miniTn5 of strain 2707 in 7178 | This study |

1. Stolz, A., Busse, H. J. & Kampfer, P. *Pseudomonas knackmussii* sp. nov. *Int J Syst Evol Microbiol* **57**, 572-576 (2007).

2. McClure, N. C., Weightman, A. J. & Fry, J. C. Survival of *Pseudomonas* *putida* UWC1 containing cloned catabolic genes in a model activated-sludge unit. *Appl Environ Microbiol* **55**, 2627-2634 (1989).

3. Sentchilo, V. S., Ravatn, R., Werlen, C., Zehnder, A. J. B. & van der Meer, J. R. Unusual integrase gene expression on the *clc* genomic island of *Pseudomonas* sp. strain B13. *J Bacteriol* **185**, 4530-4538 (2003).

4. Minoia, M., Gaillard, M., Reinhard, F., Stojanov, M., Sentchilo, V. & van der Meer, J. R. Stochasticity and bistability in horizontal transfer control of a genomic island in *Pseudomonas*. *Proc Natl Acad Sci U S A* **105**, 20792-20797 (2008).

5. Miyazaki, R. & van der Meer, J. R. A dual functional origin of transfer in the ICE*clc* genomic island of *Pseudomonas knackmussii* B13. *Mol Microbiol* **79**, 743-758 (2011).

6. Carraro, N., Richard, X., Sulser, S., Delavat, F., Mazza, C. & van der Meer, J. R. An analog to digital converter controls bistable transfer competence of a widespread integrative and conjugative element. *Elife* **9** (2020).

7. Pradervand, N., Sulser, S., Delavat, F., Miyazaki, R., Lamas, I. & van der Meer, J. R. An operon of three transcriptional regulators controls horizontal gene transfer of the Integrative and Conjugative Element ICE*clc* in *Pseudomonas* *knackmussii* B13. *PLoS Genet* **10**, e1004441 (2014).
