# Supplementary material for: A bistable prokaryotic differentiation system underlying development of conjugative transfer competence
Source: PLoS Genet. 2022 Jun 28;18(6):e1010286. doi: 10.1371/journal.pgen.1010286 (PMC9286271; doi:10.1371/journal.pgen.1010286)
Supplement: S2 Table — (DOCX) [file pgen.1010286.s002.docx]

Supplementary table S2: Primers used.

| **Number** | **Sequence 5’-3’** | **Target** | **Restriction site** | **Objective** |
| --- | --- | --- | --- | --- |
| **051005** | CAAGAAGGACCATGTGGTC | eGFP gene on pBAM | **-** | Construct verification |
| **060605** | TTTTTTGAATTCGCGCAATCACCGATCGCGCAT | *inr*R gene on ICE*clc* (94688-94708) | **-** | Amplification of inrR gene |
| **060606** | TTTTTTTCTAGAATGAGCGATCTGAACCAACCG | *inr*R gene on ICE*clc* (95201-95220) | **-** |  |
| **070418** | CAGGAAACAGCTATGACC | Universal M13 primer | **-** | Construct verification |
| **090803** | TTTTGAATTCCTTGCCAAGGTCGGGGTC | P_int_ (32-49) | *EcoRI* | *Amplification of Pint-mcherry reporter system* |
| **110404** | CTTCAGCGTCATAATGGC | mcherry gene | **-** |  |
| **111201** | TTTTTGGATCCTTCGCTGGAACAGAGAGAGCAT | *traI* of ICE*clc* (52071-52093) | *BamH*I | *Short upstream region of traI* |
| **111202** | TTTTTTCTAGAGATGCTCTCCAGAGTCCAAGAAT | *traI* of ICE*clc* (52307-52329) | *Xba*I |  |
| **111203** | TTTTTTCTAGATTCGCTGGAACAGAGAGAGCAT | *orf52324-53196* of ICE*clc* (52071-52093) | *Xba*I | *Opposite orientation* |
| **111204** | TTTTTGGATTCGATGCTCTCCAGAGTCCAAGAAT | *orf52324-53196* of ICE*clc* (52307-52329) | *BamH*I |  |
| **111205** | TTTTTGGATCCAAAGACATGGCGAACCTCCGGA | *orf53587-58432* of ICE*clc* (58925-58946) | *BamH*I | *Upstream region of 58432* |
| **150803** | TTTTTTCTAGACCTGCTTGATCGCCA | *orf53587-58432* of ICE*clc* (59252..59266) | *Xba*I |  |
| **111207** | TTTTTGGATCCCCAGATGTCCATCATTGTGCT | *orf59110-62755* of ICE*clc* (63082-63102) | *BamH*I | *Upstream region of 62755* |
| **111208** | TTTTTTCTAGAATTTCCAGCAACGAAGCCGTA | *orf59110-62755* of ICE*clc* (63383-63403) | *Xba*I |  |
| **111209** | TTTTTGGATCCGATCGTGCGGAACACCCA | *orf63176-66202* of ICE*clc* (66439-66456) | *BamH*I | *Upstream region of 66202* |
| **111210** | TTTTTTCTAGAATTGCTGGTTGTGGCGTCGAT | *orf63176-66202* of ICE*clc* (66785-66805) | *Xba*I |  |
| **111211** | TTTTTGGATCCATCACGAGAAAAGTGGGCAC | *orf66625-67231* of ICE*clc* (67562-67581) | *BamH*I | *Upstream region of 67231* |
| **111212** | TTTTTTCTAGAACATAGACCACTCAACGAGA | *orf66625-67231* of ICE*clc* (67901-67920) | *Xba*I |  |
| **111213** | TTTTTTCTAGAATCACGAGAAAAGTGGGCAC | *orf67800* of ICE*clc* (67562-67581) | *Xba*I | *Upstream region of 67800* |
| **111214** | TTTTTGGATCCACATAGACCACTCAACGAGA | *orf67800* of ICE*clc* (67901-67920) | *BamH*I |  |
| **120203** | TTTTTGGATCCGGACGGGCTCCTTGGAAAAG | *orf85934-88400* of ICE*clc* (88619-88638) | *BamH*I | *Upstream region of 88400* |
| **120204** | TTTTTTCTAGAGACCCTCATTACATCGACATGAC | *orf85934-88400* of ICE*clc* (89229-89252) | *Xba*I |  |
| **120205** | TTTTTGGATCCATGTCGGGTCTCCTGTTCGT | *orf89247-89746* of ICE*clc* (91351-91370) | *BamH*I | *Upstream region of 89746* |
| **120206** | TTTTTTCTAGATTCCTGACCCGACCCCGTTC | *orf89247-89746* of ICE*clc* (91876-91895) | *Xba*I |  |
| **120207** | TTTTTGGATCCTAATAGGACGACAACGTGGG | *orf96323-100033* of ICE*clc* (100771-100790) | *BamH*I | *Upstream region of 100033* |
| **120208** | TTTTTTCTAGATGATGCGTGCCGGCAAGTTC | *orf96323-100033* of ICE*clc* (101050-101069) | *Xba*I |  |
| **120209** | TTTTTGGATCCTTCATCGAGACGCAAGATGC | *orf100952* of ICE*clc* (101113-101132) | *BamH*I | *Upstream region of 100952* |
| **120210** | TTTTTTCTAGAATTACCGATCGCACGCTGCAA | *orf100952* of ICE*clc* (101337-101357) | *Xba*I |  |
| **120211** | TTTTTGGATCCATGCTCCGTCTCCTTCCAGGA | *orf101284* of ICE*clc* (102043-102063) | *BamH*I | *Upstream region of 101284* |
| **120212** | TTTTTTCTAGACGCAGTCGTCACAACGTCAT | *orf101284* of ICE*clc* (102655-102674) | *Xba*I |  |
| **130701** | TTTTAAGCTTCGAGGTGTGAAGGTCGAAG | *81655up_for (82531-82549)* | *HindIII* | *Upstream region for deletion of 81655-75419* |
| **130702** | TTTTTTGGATCCTGCGCATGGACAGGCCATT | *81655up_rev (83337-83355)* | *BamHI* |  |
| **130703** | TTTTTTTCTAGATCAATCCCGAGCCAGCTTC | *75419down_for (74442-74460)* | *XbaI* | *Downstream region for deletion of 81655-75419* |
| **130704** | TTTTTAAGCTTCGCCGCCGGTTTCTTGTTA | *75419down_rev (75383-75401)* | *HindIII* |  |
| **130705** | TTTTAAGCTTGAAACTCTCCTTGGGATAG | *74436up_for (75312-75330)* | *HindIII* | *Upstream region for deletion of 74436-68241* |
| **130706** | TTTTTTGGATCCTCAAGGACATCGGTGGCAA | 74436up_rev (76230-76248) | BamHI |  |
| **130707** | TTTTTTTCTAGACAGCCGCGGGTAATCGAAG | *68241down_for (67390-67408)* | *XbaI* | *Downstream region for deletion of 74436-68241* |
| **130708** | TTTTTTAAGCTTACCGGCAGCGCAGGAAGTA | 68241down_rev (68219-68237) | HindIII |  |
| **210611** | AGGGATAACAGGGTAATCTGAATTCCCCACCAGTTCGCCTCGGCAT | *D88400-84388UP.f* (89622-89642) | EcoRI | *Upstream region for deletion of 88400-84388* |
| **210612** | TCAGGCTTGCTGGGACATGGACGGGCT | *D88400-84388UP.r* (88604-88627) | *-* |  |
| **210613** | AGCCCGTCCATGTCCCAGCAAGCCTGAAGCAGCAGCACGTCCTGCTCA | *D88400-84388DW.f* (84323-84343) | *-* | *Downstream region for deletion of 88400-84388* |
| **210614** | GAAGCTTGCATGCCTGCAGGTCGACGGAGTTGCAGCCGTGGCGCGAT | *D88400-84388DW.r* (83314-8335) | SalI |  |
| **200901** | AATCAGAATTCGAGCTCGCCCTAGTGGCGGGCCGACCGGGA | *PtraI.F* (52104-52123) | *-* | *Long upstream region of traI* |
| **200903** | TTCGAGGCATGCCTGCAGCCCCGATCACACAGTCGTGGAA | *PtraI.R2* (52743-52761) | *-* |  |
| **210615** | AATCAGAATTCGAGCTCGCCCGAGGAAGGACAAGGGATTCC | *P84835.f* (85666-85685) | *-* | *Upstream region of 84835* |
| **210616** | TTCGAGGCATGCCTGCAGCCCGCGCCACGCACCATTCTTCCT | *P84835.R2* (86290-86310) | *-* |  |
| **210617** | AATCAGAATTCGAGCTCGCCCGAAATCGACCGGGTGGCGACAT | *P89247.f* (89517-89538) | *-* | *Upstream region of 89247* |
| **210618** | TTCGAGGCATGCCTGCAGCCCCAAGGCAATCGGCAATTCCAT | *P89247.r* (89830-89850) | *-* |  |
| **210619** | AATCAGAATTCGAGCTCGCCCCTCTCGGGCCACGAGCGGCA | *P73676.f* (74320-74339) | *-* | *Upstream region of 73676* |
| **210620** | TTCGAGGCATGCCTGCAGCCCGTGGACGCCTGGTCGATCGAT | *P73676.r* (75123-75143) | *-* |  |
