# Supplementary material for: A bistable prokaryotic differentiation system underlying development of conjugative transfer competence
Source: PLoS Genet. 2022 Jun 28;18(6):e1010286. doi: 10.1371/journal.pgen.1010286 (PMC9286271; doi:10.1371/journal.pgen.1010286)
Supplement: S1 Fig — A General overview of the ICE core region. B Individual selected promoter/upstream fragments and their sizes (small cap letters corresponding to fragment indications in the main text). (PDF) [file pgen.1010286.s003.pdf]

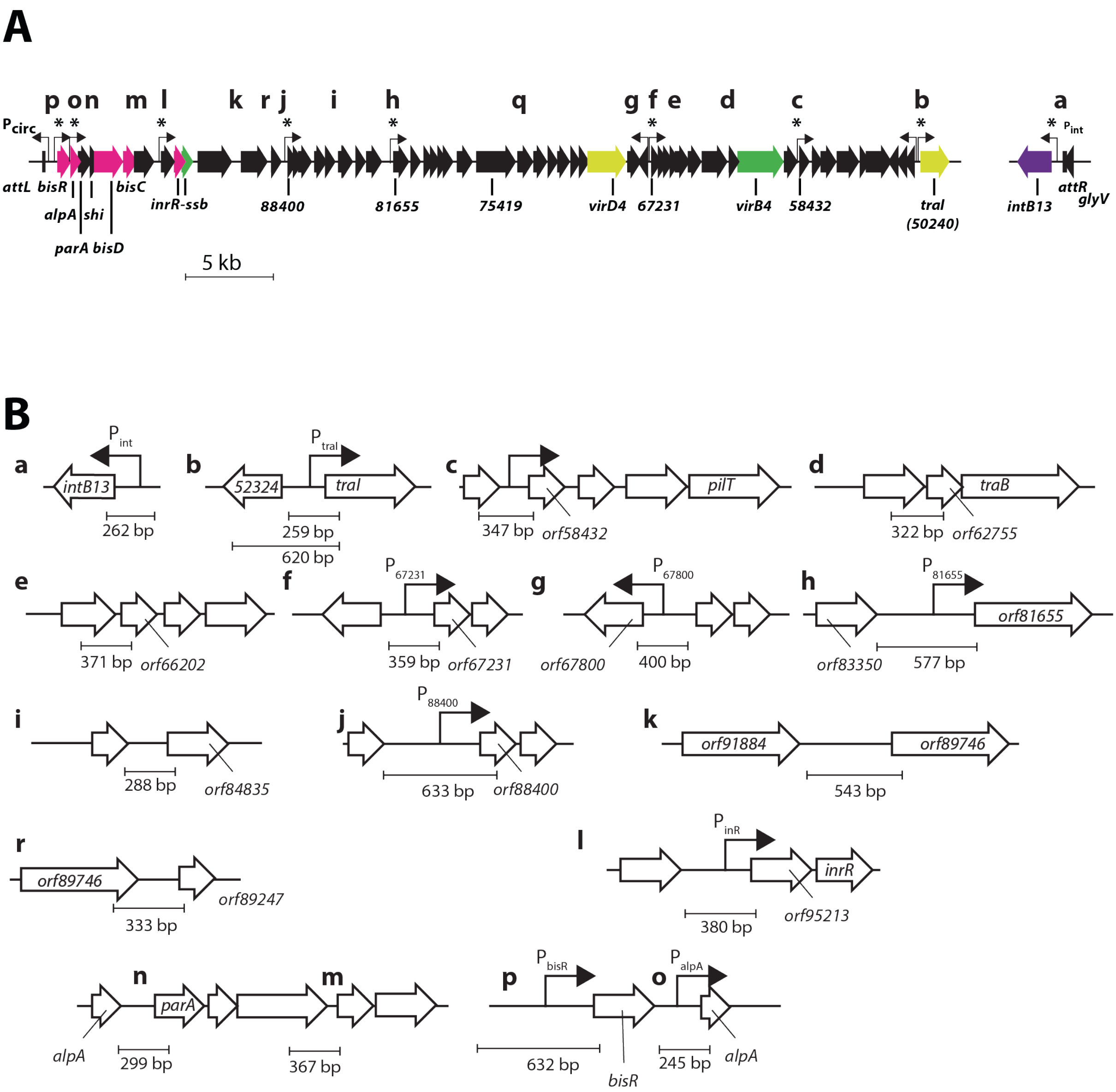

Supplementary figure 1. Cloned and tested upstream/promoter fragments of ICE core genes.

A General overview of the ICE core region. **B** Individual selected promoter/upstream fragments and their sizes (small cap letters corresponding to fragment indications in the main text).
