# Supplementary material for: A bistable prokaryotic differentiation system underlying development of conjugative transfer competence
Source: PLoS Genet. 2022 Jun 28;18(6):e1010286. doi: 10.1371/journal.pgen.1010286 (PMC9286271; doi:10.1371/journal.pgen.1010286)
Supplement: S2 Fig — A) Plots show regions tested for promoter activity with read coverage per basepair position from RNA-seq (for a single representative replicate) at the indicated conditions (3CBA, exponential phase in black; stationary phase in green), plotted for the relevant P. putida genome region with the integrated ICEclc on the x-axis (in Mbp). B) Read coverage of ICEclc transcripts in P. putida ICEclc in stationary phase conditions after growth with 3CBA (green) or succinate (brown) as carbon substrate. Blue lettered bars point to cloned fragments tested for promoter activity at single cell level. Dotted black arrows point to subpopulation-dependent tc cell promoters; straight lines when expressed in all cells. Open directional bars (< or >) correspond to relevant coding regions on ICEclc. Pcirc, outward facing constitutive promoter. (PDF) [file pgen.1010286.s004.pdf]

A 3-CBA expo vs stat

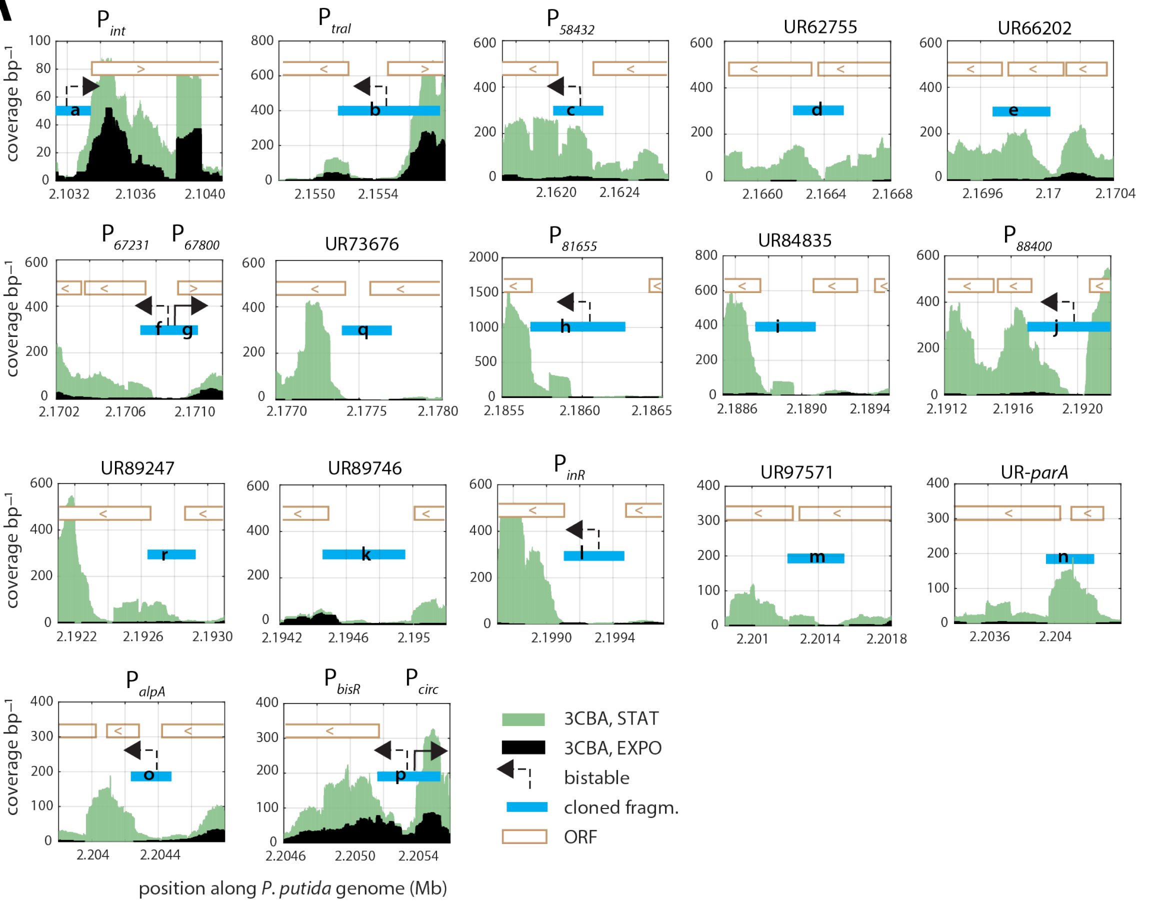

B 3-CBA stat vs Succinate stat

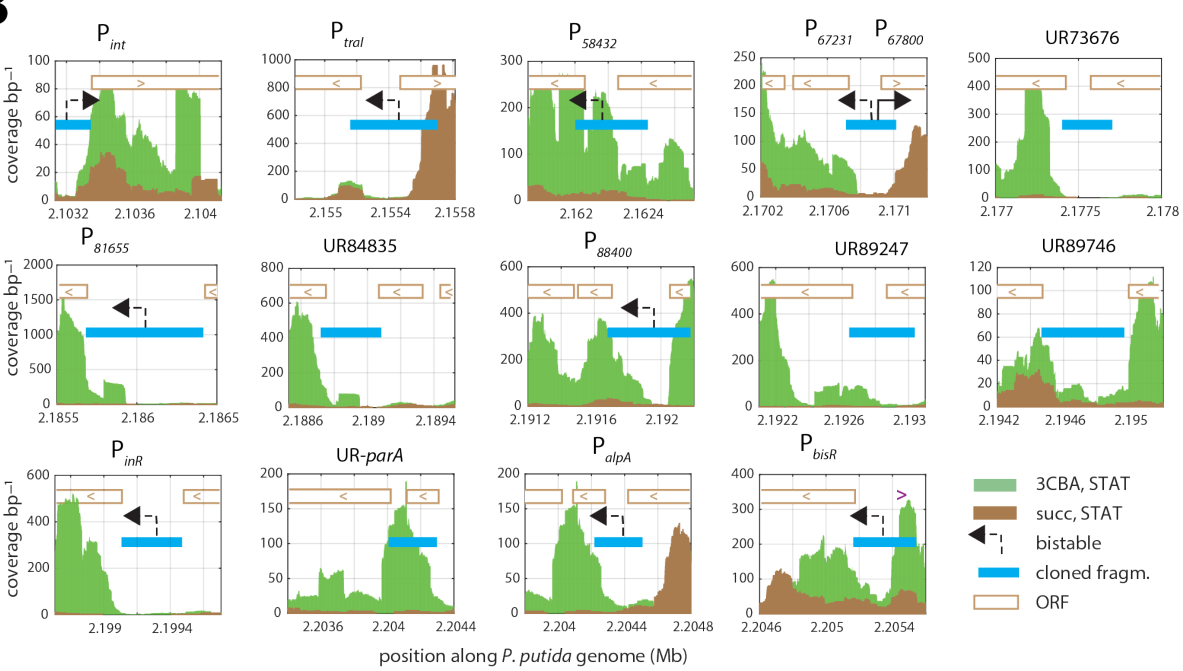

Supplementary figure 2. Read coverage of ICE*clc* transcription in *P. putida*.

A Plots show regions tested for promoter activity with read coverage per basepair position from RNA-seq (for a single representative replicate) at the indicated conditions (3CBA, exponential phase in black; stationary phase in green), plotted for the relevant *P. putida* genome region with the integrated ICEclc on the x-axis (in Mbp).

B Read coverage of ICE*clc* transcripts in *P. putida* ICE*clc* in stationary phase conditions after growth with 3CBA (green) or succinate (brown) as carbon substrate.

Blue lettered bars point to cloned fragments tested for promoter activity at single cell level. Dotted black arrows point to subpopulation-dependent tc cell promoters; straight lines when expressed in all cells. Open directional bars (< or >) correspond to relevant coding regions on ICE*clc*. Pcirc, outward facing constitutive promoter.
