# Supplementary material for: A bistable prokaryotic differentiation system underlying development of conjugative transfer competence
Source: PLoS Genet. 2022 Jun 28;18(6):e1010286. doi: 10.1371/journal.pgen.1010286 (PMC9286271; doi:10.1371/journal.pgen.1010286)
Supplement: S3 Fig — Motif identified by MEME. Sequence of PtraI added manually to align.No other similar motif was found on ICEclc. Distance indicated to the start codon of the downstream gene. Distance to the mapped transcription start site in the PinR-promoter: 152 bp. (PDF) [file pgen.1010286.s005.pdf]

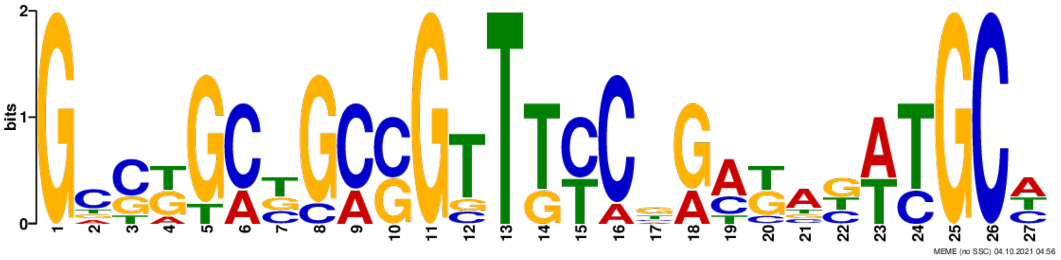

promoter		motif		Distance to start	e-value
PalpA	GCCGCAAGCT	GCGGGCCGCCGTTTCCTGATATTTGCA	AGCCCGAATC	-111bp	9.1e-13
PinR	ACCGCACTGC	GATGGCGCCGGTTTCAGGAGCGACGCT	GACATTCGCG	-175bp	2.8e-8
P88400	TCGGGGCTGG	GCCGGATGCCGTTTTCCAACTGACGCA	GCGCGGCCCG	-223bp	8.9e-10
P81655	CTGCCCCCAT	GCCTGCTGACGTTGTCAGCGACATGCC	AGGCAACCAT	-195bp	2.0e-10
P67231	CATGAATCCG	GTCATCTGCGGGTTCCTATTGTTTGCA	GCCTAAAGCA	- 59bp	9.8e-9
P58432	CCGCCGAAAT	GCCTGCCGACGTTGTCGGCGACATGCC	CAGGTAGTCC	-190bp	3.4e-10
PtraI	CATGAATGGC	AGGCATGGTGGTTCTCCTGATAGCGAC	ACCACTATGG	-274bp	man
Pint	ACAGGAATAC	GGGTGAGGCGGCTTCCGGATAGTTGCT	GGGGCTGAGC	- 59bp	7.4e-10

Supplementary figure 3. **Common sequence motif in the identified transfer competence promoters of ICE*clc*.** Motif identified by MEME [1]. Sequence of P_{traI} added manually to align.
No other similar motif was found on ICE*clc*. Distance indicated to the start codon of the downstream gene. Distance to the mapped transcription start site in the P_{inR}-promoter [2]: 152 bp.

1) Bailey TL, Boden M, Buske FA, Frith M, Grant CE, Clementi L, et al. MEME SUITE: tools for motif discovery and searching. Nucleic Acids Res. 2009;37(Web Server issue):W202-8. doi: 10.1093/nar/gkp335.
2) Minoia M, Gaillard M, Reinhard F, Stojanov M, Sentchilo V, van der Meer JR. Stochasticity and bistability in horizontal transfer control of a genomic island in *Pseudomonas*. Proc Natl Acad Sci U S A. 2008;105(52):20792-7.
