# Supplementary material for: A bistable prokaryotic differentiation system underlying development of conjugative transfer competence
Source: PLoS Genet. 2022 Jun 28;18(6):e1010286. doi: 10.1371/journal.pgen.1010286 (PMC9286271; doi:10.1371/journal.pgen.1010286)
Supplement: S4 Fig — Single copy inserted promoter-reporter fusion pairs as indicated on top of each panel, with color legend. Left panels show global increase of the inferred tc cell population from the respective marker. The right panels show paired automatically detected onsets of fluorescence increase in individual tc cells. Values of 1 are artificially attributed when no slope was detected for the respective cell and fluorescence reporter. See further, explanation to Fig 5 in main text. Note that panel D global increase could not be quantified because of drifting image focus between time 19 and 20 h. (PDF) [file pgen.1010286.s006.pdf]

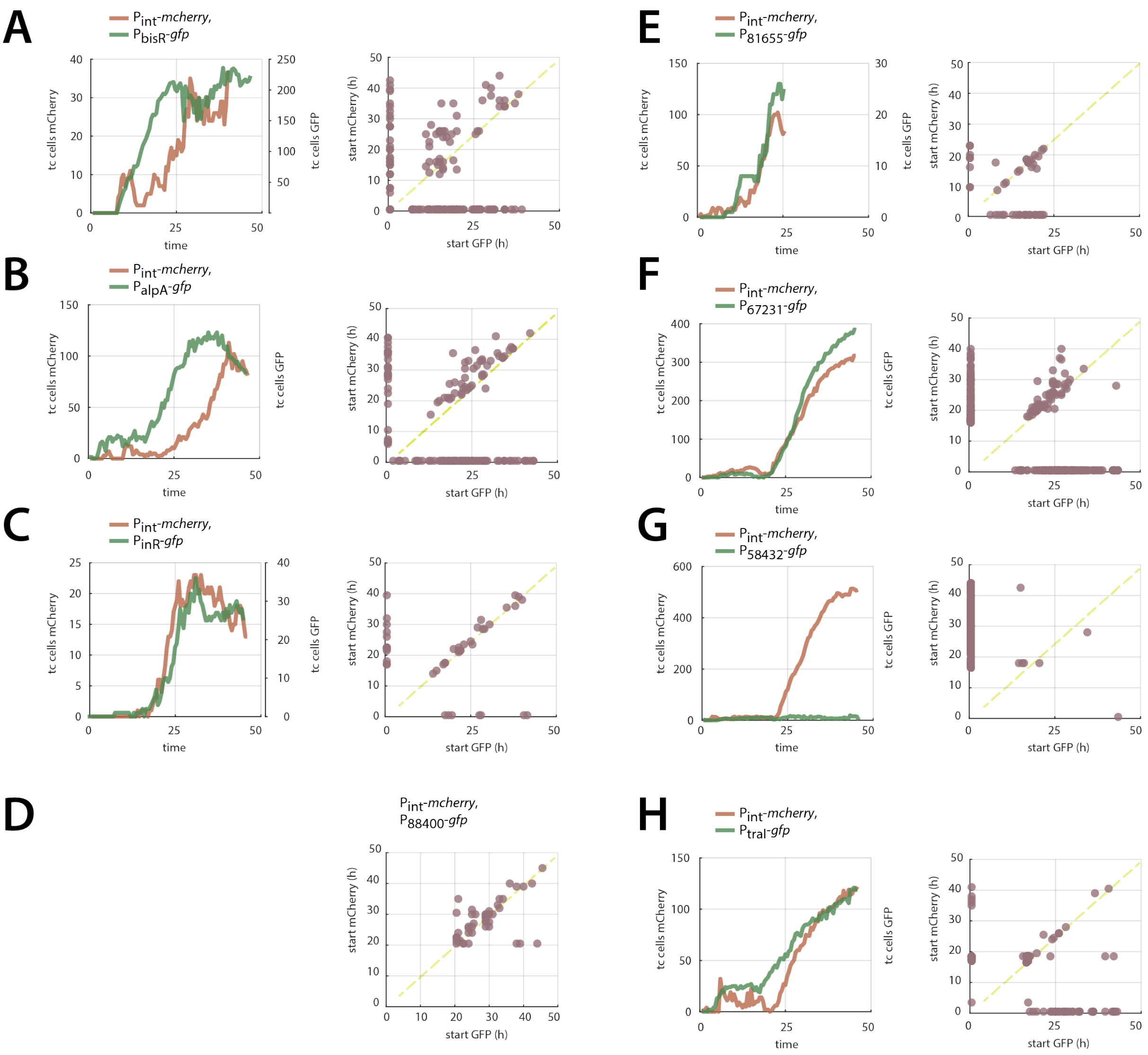

Supplementary figure 4. Timing of onset of transfer competence promoter expression in *P. knackmussii* (A-G) and *P. putida* ICE*clc* (H). Single copy inserted promoter-reporter fusion pairs as indicated on top of each panel, with color legend. Left panels show global increase of the inferred tc cell population from the respective marker. The right panels show paired automatically detected onsets of fluorescence increase in individual tc cells. Values of 1 are artificially attributed when no slope was detected for the respective cell and fluorescence reporter. See further, explanation to Figure 5 in main text. Note that panel D global increase could not be quantified because of drifting image focus between time 19 and 20 h.
