# Supplementary material for: A bistable prokaryotic differentiation system underlying development of conjugative transfer competence
Source: PLoS Genet. 2022 Jun 28;18(6):e1010286. doi: 10.1371/journal.pgen.1010286 (PMC9286271; doi:10.1371/journal.pgen.1010286)
Supplement: S5 Fig — Cells sampled in stationary phase after growth on succinate. Comparisons are the same P. putida reporter strains but with empty plasmid (pME6032). Cell fluorescence distributions are plotted as their expected versus observed quantile; each plot showing a single biological replicate with independent reporter gene insertion position, grouped from n = 10 images per sample. Each dot corresponds to a single segmented cell observation. Note the strongly tailed distributions for some constructs. (PDF) [file pgen.1010286.s007.pdf]

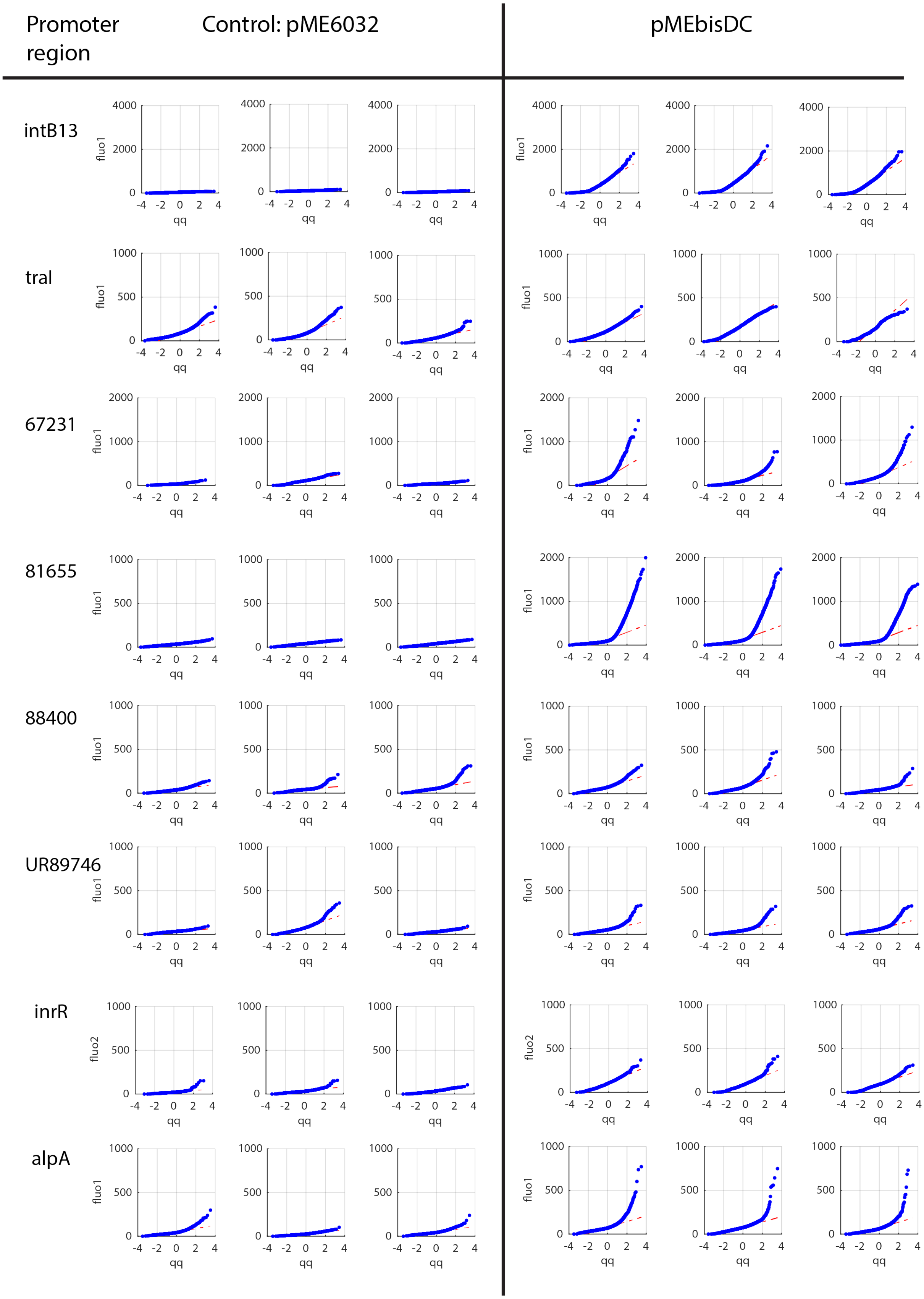
Promoter region
Control: pME6032
pMEbisDC
intB13
traI
67231
81655
88400
UR89746
inrR
alpA
fluo1
fluo2
qq

Supplementary figure 5. Cell fluorescence distribution from indicated single copy promoter-reporter fusions in *P. putida* without ICE*clc*, but induced or not for production of the BisDC activator complex (pMEbisDC).

Cells sampled in stationary phase after growth on succinate. Comparisons are the same *P. putida* reporter strains but with empty plasmid (pME6032). Cell fluorescence distributions are plotted as their expected versus observed quantile; each plot showing a single biological replicate with independent reporter gene insertion position, grouped from n=10 images per sample. Each dot corresponds to a single segmented cell observation. Note the strongly tailed distributions for some constructs.
