# Supplementary material for: A bistable prokaryotic differentiation system underlying development of conjugative transfer competence
Source: PLoS Genet. 2022 Jun 28;18(6):e1010286. doi: 10.1371/journal.pgen.1010286 (PMC9286271; doi:10.1371/journal.pgen.1010286)
Supplement: S6 Fig — Regions are aligned to the gene cluster containing inrR and ssb (ochre), and then emphasize the conserved regions with unknown functions orf88400—orf81655. Ortholog genes are colored similarly. Arrows indicate the corresponding open reading frame length and orientation. Numbers below represent the percent nucleotide identity to ICEclc. White open arrows point to open reading frames not generally conserved with ICEclc. (PDF) [file pgen.1010286.s008.pdf]

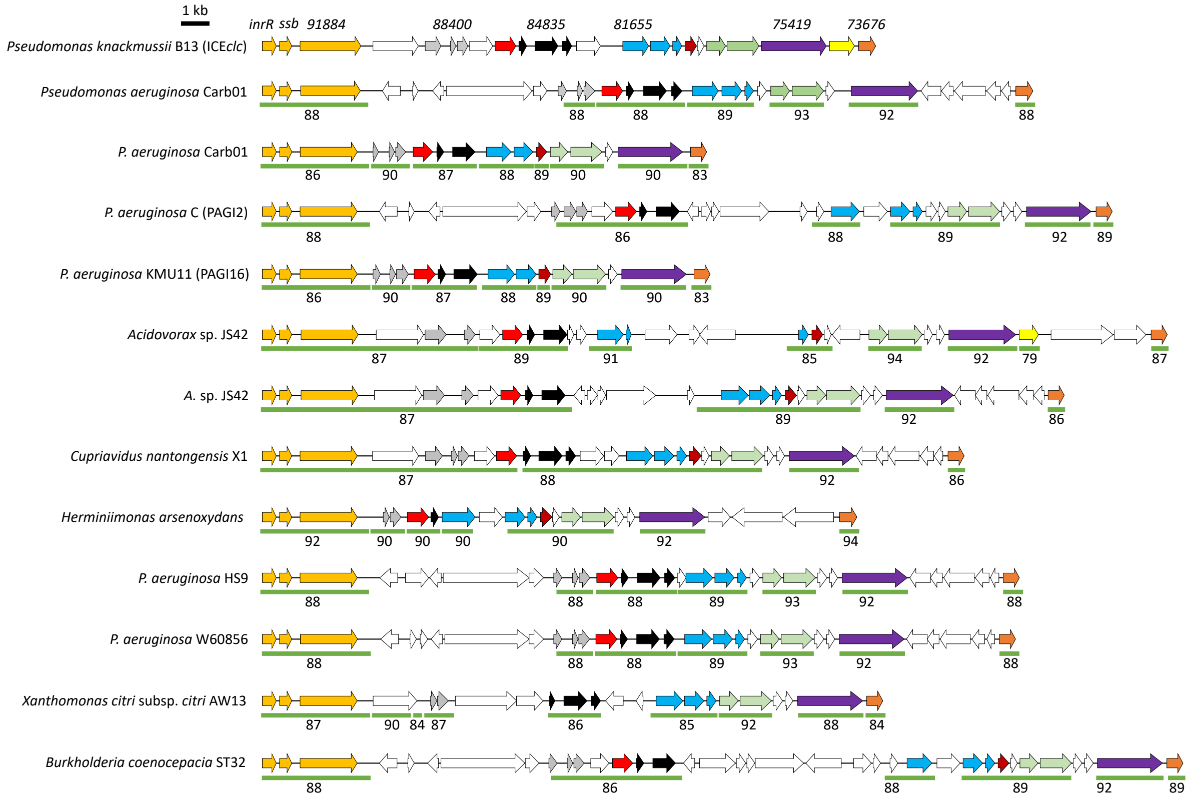

Supplementary figure 6. ICE*clc* gene and gene synteny conservation to putative ICE in genomes of other Gamma- and Betaproteobacteria.

Regions are aligned to the gene cluster containing *inrR* and *ssb* (ochre), and then emphasize the conserved regions with unknown functions *orf88400* – *orf81655*. Ortholog genes are colored similarly. Arrows indicate the corresponding open reading frame length and orientation. Numbers below represent the percent nucleotide identity to ICE*clc*. White open arrows point to open reading frames not generally conserved with ICE*clc*.
