# Supplementary material for: A bistable prokaryotic differentiation system underlying development of conjugative transfer competence
Source: PLoS Genet. 2022 Jun 28;18(6):e1010286. doi: 10.1371/journal.pgen.1010286 (PMC9286271; doi:10.1371/journal.pgen.1010286)
Supplement: Attachment — Submitted filename: Reply_Reviewers.docx [file pgen.1010286.s014.docx]

**Author’s reply and actions to Reviewers’ comments**

Please note that the reference to page and line numbers is with respect to the PDF with all changes highlighted.

**Comments to the Authors:**

Reviewer #1: The manuscript by Sulser et al. describes how six gene clusters of an integrative conjugative element (ICE) are activated in a small subset of the cells (<10%). By using a dual labelling strategy (GFP/mCherry) they show that in most of these clusters, the subset of cells that activate transcription correspond to cells transcribing the integrase gene. These results suggests that activation of the ICE promoters is correlated with integrase expression, and ICE conjugation. Previous results have shown a bimodal activation pattern of ICE conjugation: upon entrance in stationary phase, only a small fraction of the cells actively transcribe certain key transcriptional activators (BisR, InR), which result in transfer competence. By looking at the expression levels of the ICE promoters in different mutants, the authors show that these genes constitute the final part of the activation program leading to ICE conjugation. Overall, the paper is well written and experimentally sound. Results presented here contribute to completing the picture of the ICE activation program, thus these findings are of broad interest to understanding the dynamics and physiology of these mobile genetic elements. My only concerns refer to the use of certain terminology and data presentation issues, which I think should be addressed for a better, more rigorous presentation of results.

*Reply : We thank the reviewer for the concise summary of the work and the positive general appreciation.*

Minor Comments

1.- My main concern is the rather lax usage of two terms: bistable and orthogonal. From the title to the discussion, these two buzzwords are used frequently in the paper, yet I am afraid their employment is not entirely justified by results presented here. A bimodal distribution is not the equivalent of a bistable system. The latter tends to produce (but not always) the former, and bimodality may be achieved by different mechanisms that do not necessarily imply bistability. I have no objection to mention the possibility of this being an example of a bistable system in the discussion -it may well be one- but results presented do not justify the claim of the title. I might be missing something, but in the references cited by the authors there is neither much evidence of the usual hallmarks of bistability (positive feedback, hysteresis, etc…) nor any stability analysis proper, which could justify the claim. The strongest evidence of a possible bistable mechanism is the cell arrest phenotype demonstrated in Ref18. However as shown in figure 1C of the manuscript, the promoter for parA/Shi, responsible for cell arrest, is not bimodal. I am also a puzzled by the usage of ref 42 as a justification for bistable behavior. This paper from Leibler mostly deals with stochastic switching as an alternative to deterministic switching (for example by a bistable activator). If anything, it is a demonstration of how other mechanisms may produce bimodal distributions. Confusion between bistability and bimodality is widespread in the literature, thus this may sound like a finicky request. However, the differences are important for people working in the dynamics of regulatory systems. I have also the feeling that some authors tend to use bistable because they perceive this as a more “sophisticated” or valuable mechanism. It is not: many bistable systems never achieve bimodaility. I would suggest the authors to adhere to bimodality, which I think is the most correct term describing their results. Indeed, one possible mechanistic explanation for their results could lie in two critical regulators being stochastically activated and working in a concerted AND fashion (BisR + InrR? or maybe RpoS?). This could result in a bimodal distribution without the need of a positive feedback loop or any other bistable behavior.

*Reply: We apologize but it was not our intention to use the terms ‘bistability’ and ‘orthogonality’ in a lax manner; nor to make the work look more ‘sophisticated’. Rather the contrary, although apparently we have not conveyed this properly.*

*We follow here the definitions of bistability as proposed in Dubnau and Losick 2006 (‘Bistability in bacteria’) : ‘..the population bifurcates into subpopulations..’ ‘Bistability implies that a regulatory system can switch between two alternative states but cannot rest at intermediate states’. This is similar to the definition by Siegal-Gaskins et al. (PLoS Comput Biol 2010) ‘Bistability–the coexistence of two stable equilibria in a dynamical system–is responsible for the switch-like behavior seen in a wide variety of cell biological networks, such as those involved in signal transduction [1], cell fate specification [2–4], cell cycle regulation [5], apoptosis [6–8], and in regulating extracellularDNA uptake (competence development) [9].’ These authors further suggested that mathematical tools and modeling can help to determine whether a particular network is bistable.*

*Our arguments for the existence of a bistable program of the ICE (rather than a collection of bimodally expressed promoters) are the following: (i) the regulatory architecture is sufficient for causing and maintaining bistability (i.e., double positive feedback), as empirically shown, mathematically modeled and explained in Ref.26; (ii) the biological evidence for separate alternative states; one being the ICE in a non-active integrated state, the other the ICE going through its program of horizontal transmission in dedicated cells, and (iii) different but defined end-states: i.e., transfer competent cells arresting cell division and not returning to the ICE inactive state (Ref. 18 and 19); and non-tc cells continuing cell division. This would – to our understanding, fulfill the ‘requirements’ of being bistable; and is appropriate to describe the specific development program exerted by the ICE in transfer competent cells.*

*The promoter for the parA/shi-genes is the alpA-promoter, which we show is bimodal. This must be a misunderstanding.*

*We used reference 42 to illustrate the phenomena of phenotypic switching.*

*Action: We clarified this on p. 5, l.18-25 by introducing the proposed hierarchical network earlier in the text (upon suggestion by reviewer 2). We use ‘bimodal’ rather than ‘bistable’ until the discussion, where we list the arguments to conclude that the transfer competence pathway must be bistable (p.15, l.19-25).*

The second term that I think should be used with caution is "orthogonal". In particular, I would suggest the authors to clearly state which two aspects/things are being considered orthogonal when the term is used. Otherwise the wording results confusing, as for example in the abstract. There, the authors speak of “six gene clusters coordinately and orthogonally expressed in the same cell subpopulation”. Yet this is patently absurd: if they are coordinated they cannot be orthogonal to each other! My guess is that the authors were trying to indicate that these genes are orthogonal to the host regulatory network. This may well be the case, but I find it difficult to prove, given that RpoS, as indicated by the authors, is a key player in the circuit. Orthogonal just merely indicates that two variables are independent of each other, but this most often gives little to no insight. Moreover, in this case results actually may hint at the opposite. As shown in Figure 6 the activation dynamics is quite different in cells growing in exponential phase, compared to stationary cultures. This could be caused just by the effects of protein dilution/concentration in growing/arrested cells (thus a circuit truly orthogonal). However this could be also caused by RpoS activation, or any other regulator of the stationary phase. From the data presented here, I don´t think we can favor any of the two options.

*Reply : We thank the reviewer for these suggestions. Indeed, in the abstract we referred to being orthogonal with respect to the host. What we were trying to convey here is the message that the ICE is executing a program that is within a host cell but that needs to be limited to a small proportion of cells in a population. The program therefore needs to be properly shielded from unnecessary noise and activation in non-target cells – as we mentioned on p. 14, l. 19-26. We believe this justifies the terminology of orthogonality; although we agree that the term may be confusing as well.*

*We raised the concept because it permits to look for the factors that actually help selectively activating certain promoters and shielding others. In contrast to bacteriophage orthogonal expression factors, the ones from ICE may be selective to the subpopulation of initiated cells.*

*RpoS is important for activation of the PinR-promoter in stationary phase, and, as such, may link the ICE network to the host. However, since RpoS (protein) levels are uniformly distributed in stationary phase cells (see Ref. 32, where we measured this using translational fluorescent protein fusions), we find it hard to understand how that would cause orthogonality of the system. This would require specific ICE proteins.*

*Action : We refrained from using the term orthogonality in the title and in the introductory text – and rewrote the discussion to clarify the concept of how ICE gene expression might remain restricted to a subpopulation of tc cells (p.16, l.14-26).*

2.- I really liked the quartile plots in figure 2, which clearly show the activation of a fraction of the population beyond the statistical expectation, as judged from the rest of the distribution. However I found panel 4d a bit clumsy. My guess is that the authors were trying to show the level of induction in the “active” population, compared to the inactive population. Thus they show the averages in both sets. The problem is that , since the active population is defined as the set of cells in the upper quartile of the distribution, the reasoning becomes circular. The average of the upper quartile of any distribution is always higher than the average of the lower quartiles, by definition. To show the information that the authors want to convey here , one alternative could be to show the distribution , deconvolve, and calculate the median/mode of each subpopulation. Alternatively, and probably much easier, the authors could just show the distribution on an x scale, showing the quartiles, so the reader may have an intuition of the magnitude of the increase in the "active" population.

*Reply : We thank the reviewer here. Yes, indeed, there is some circular reasoning here – but panel 4d was really made to show replicate variation on independent insertion clones and allow statistical comparison of expression levels (which is hard to do from a single qq-plot). So, whereas the reviewer is right that by definition, the median of the upper quartile levels is higher than that of the lower; this is not necessarily the case when comparing different clones of the same genetic construct. As a reminder, because we are mostly using single copy reporter gene insertions, we compare expression from at least three different clones. For this reason, we used a comparison of the averages of those – and corrected for differences in background fluorescence of images; to be correct.*

*Action: This was emphasized on p. 7, l. 10-14; and in the legend to Figure 3. We think that this does not further require changes in panel 3d or its description.*

3.- I am also a bit puzzled by figure 4. The distribution of Pint levels (measured from mCherry) should be more or less the same in all panels, right? However the scale on the y axis in the figure suggests otherwise (Pint goes from 90 to 160 when measured with P88400, but from 400 to 1400 when measured with P58432). Are the exposure times / lamp intensities changing between experiments? Does this affect to the definition of Tc competent cell? (my guess is not, because it is quartile-based, but it should be stated in the text).

*Reply : True – we thank the reviewer for this point, which was also raised by the other reviewers. The insertion of the Pint-mCHE fusion is the same in all strains, except for the d-combination and g (which is in P. putida). Exposure times were kept the same, but, indeed, two camera systems were used as our data collection spans several years here. Further, as we discuss on p. 15, l.6-14, we think there is variation in the expression of the Pint-mCHE fusion, which is due to noise effects originating from having promoter pairs (see, for example, the new time-lapse series of figure 5). If one would have low cell titers of relevant transcription factors, then it would actually matter if these TFs are distributed over one operator (the native ICE), plus an additional one with the Tn7 insertion (Pint-mCHE), or with a third single copy Tn5 insertion (the tester promoter in Fig. 4).*

*Action: We have corrected the text on p. 20, l. 21-26 to clarify qq-plot analysis, and explained that this makes tc-cell subpopulation determination insensitive to differences in fluorescence intensity. To avoid confusion, we chose to display normalized fluorescence data in the revised Figure 4.*

4.- I also found figure 5 a bit confusing. Panel D, for example, gives little information , thus I think it may be better suited for supplementary material. Panel A, as it is now, is really hard to decipher because the high number of cell traces included. I am not entirely sure how this could be improved, but I would recommend the authors to reduce the burden of this panel. This could be achieved by only including cell trajectories that do show activation ( we already know that up to 90% are going to never activate). From the subset of cells that activate, I would pick a representative sample (maybe 5-10) and plot them in different colors. This way we may be able to check whether the cell that quickly fires in the Pint-mCherry promoter increases simultaneously, earlier or later the GFP trace. Regarding the temporality of activation, I would also recommend caution in the interpretation: mCherry matures in 15-30 minutes in E.coli , while the GFP does in 3-7 minutes. These differences may blur a co-activation temporal patterns. Also, some terms deserve better explanation. For example how is “start time” defined. Also, in panel D the GFP/mCherry counts are not well explained, do they correspond to the “start time”, the maximum level?

*Reply : We thank the reviewer for the comment and we agree that time-lapse presentations of tc cell development and reporter gene expression are not the most simply to display. We also agree with the reviewer (and we are aware of this) that GFP and mCHE don’t mature at the same rate. We further acknowledge that we don’t know the rates of reporter protein degradation in tc cells of* P. putida and P. knackmussii*.*

*Action: Upon suggestion of the reviewers, we revisited and repeated the time-lapse experiments for all clones. We now display only 10-20 different individual tc cell time profiles, with corresponding colours for individual cells between the two reporter signals, and we omitted the non-tc cell profiles. This is displayed in the revised Figure 5, panel A.*

*We now calculate the start timing in two different ways, as described on p. 22, l. 9-25. From the overall increase of tc cells over time (defined from qq-plotting at every time point), we can determine a subpopulation average (displayed in Fig. 5 panel B). We then further compare expression starts from individual tc cells with paired fluorescence (panel C). Here the starting time for each tc cell and fluorescence channel (GFP or mCherry) is calculated as the minimum time point where the linear regression of the fluorescence intensities over five consecutive time points is 2x higher than the average of the background (in other words: where the curve points ‘upwards’). We have removed the comparison of paired promoter expression intensities, because this is visible within traces of panel A already. Figure 5 legends and the time-lapse results sections on p.10, l.10 -p.11, l. 23 were updated accordingly.*

*The improved data analysis shows now more clearly how the bisR and alpA promoters are active earlier than the Pint- and other ‘downstream’ promoters in the proposed network hierarchy, which we explain and discuss on p. 17, l. 17-20. Limitations of qq-plot analysis are explained on p.15, l.5-15.*

Reviewer #2: This manuscript builds on previous work characterizing regulation of ICEclc and formation of a transfer competent (tc) subpopulation of cells of Pseudomonas species. It presents a large amount of work and analyses, including single cell analyses of expression of several promoters in ICEclc and RNA-seq analysis of cells in stationary phase, a subset of which are expressing ICEclc.

The topic is interesting and important. However, I found it quite difficult to determine what is new information from what was previously known. My sense is that many of the new experiments confirm previous findings obtained by somewhat different experimental approaches.

*Reply: we thank the reviewer for the positive comments. The novelty of the information presented here is that we describe the extent and coordination of the bistable regulon that controls transfer competence development, instead of having only two marker promoters (Pint and PinR). This is specified in the abstract, l. 10-18. We also rewrote the introduction on p.5, l.1-10 to make this more clear.*

From what I can tell, the novel findings here appear to be that nine promoters in ICEclc are all expressed in transfer competent (tc) cells and at least six of these appear to have coordinate regulation in stationary phase. Previously identified transcripts were tested and found to have bimodal expression in stationary phase. The gene for the regulator BisR was found to be expressed in twice as many cells in the population than ultimately commit to being transfer competent. Additionally, expression from all six promoters was dependent on the previously identified regulators InrR and BisDC. Expression from each promoter was measured in combination with expression from Pint in the same cells, demonstrating that they all turn on in the roughly the same subpopulation of cells in stationary phase, and roughly the same time. They infer that these promoters are part of the same transfer competent regulon.

*Reply: We thank the reviewer for the summary. This is mostly correct, indeed; except that the bisR and alpA promoters are activated before the others.*

*No further action needed here.*

I had to read this manuscript several times to understand and extract the main points and determine which were the key experiments. There were many experiments presented, and at times they seemed disjointed. I think some serious editing could make this clearer for readers not intimately familiar with ICEclc.

*Reply : We apologize if the main information was too hard to extract – partly because of the complexity of the experiments and partly because of the non-trivial nature of describing the unusual system and transfer competence pathway development in a subset of cells only.*

*Action: We have revisited the complete text to improve its readability.*

1. Regarding the apparently coordinate temporal control of the six promoters that are expressed in tc cells; It is clear that they are expressed in the same cell type, and not so much in others. I think we knew from first principles that if they are required for conjugation, then they must be expressed in the same cells. What seems new is that expression is largely limited to the tc cells with little expression in the non-tc population.

*Reply : We agree to this. However, in hindsight (or, as the reviewer states, ‘from first principles’) this is easy to postulate, but a priori not necessarily the case. For example, the regulatory network could be wired differently, involving post-transcriptional processes to decide which cells are conjugating or not. Furthermore, the importance of many genes on the ICE remains unclear, despite being coordinatedly expressed in tc cells. Finally, transcriptional noise and ‘shielding’ may be important factors that are not immediately guaranteed in such a system. Hence, our initial title and the use of orthogonality.*

*Action: We rewrote the introduction on p.5, l.13- p.6, l.12 to better explain the current state of affairs and the study aims.*

2. What is the resolution for determining coordination? If a gene in one of the transcription units was an activator of one of the other transcription units, is there the temporal resolution to see what could be an offset in transcription of 5-15 minutes (or less)?

*Reply : We thank the reviewer for the interesting question here. Given time lapse steps, our resolution is 30 minutes (which is longer than the fluorescent reporter maturation times). However, on top of that, single cell responses are very noisy (e.g., Fig. 5E – median response at 24 h, but with a spread of 10 h).*

*Action : We improved the temporal resolution by re-analysing and re-running more time-lapse experiments and including a further method that looks at the timing of tc cell appearance. This is independent of the time-lapse intervals, because of stochastic appearance of tc cells in the stationary phase cell population. Therefore, we can conclude now more confidently that – compared to the expression of the integrase promoter, the bisR and alpA promoters express ‘earlier’; confirming their hierarchy upstream in the regulatory network.*

*See remarks above to reviewer 1. We completely revised Figure 5. We added a new timing calculation, as described on p. 22, l.9-26. New Figure 5 legends and the time-lapse results sections on p.10, l.7 -p.11, l. 19; new discussion on network hierarchy on p. 17, l. 17-20. Limitations of qq-plot analysis are explained on p.15, l.5-14.*

3. It is not clear that these six genes are all part of the same regulon, at least directly. They all require BisDC and InrR, but there could be a hierarchy (see above).

*Reply : We thank the reviewer for bringing up this point. Indeed, there is a hierarchy, that starts with the cascade of TciR and BisR, which activates alpA-bisDC and inrR (new Fig. 2). We show that the downstream promoters can all be activated by ectopic expression of BisDC in absence of other ICE factors. It is true that activation of such promoters by BisDC is higher in presence of the ICE, suggesting either some imbalance in transcription factors or additional regulatory genes. Formally, we believe that this would qualify as being part of the transfer competence regulon (which refers to the hierarchy and not to a single operon). Take, for example, the sigma-S regulon. We have no evidence for further regulatory factors; but neither can this be excluded at this point.*

*Action: We rephrased the text on p.5, l.13-26 and p.14, l.16-24 to clarify what we mean with the ICE regulatory network. We have placed Fig.2 with the proposed model in the introduction to refer to this from the beginning.*

4. Regarding InrR and BisDC. Clearly both are needed. Could the only role of InrR be to activate BisDC? It seems that overproduction of BisDC activates gene expression in the absence of ICEclc (no inrR), perhaps bypassing the need for InrR?

*Reply : We thank the reviewer for the suggestion. The nature of InrR is unfortunately not clear and the only thing we established so far is that it is not a DNA binding protein. Overproducing BisDC in absence of the ICE indeed activates the target promoters (Fig. 6), although not to the level of the complete ICEclc. Also, deletion and copy numbers of inrR are ‘modulating’ inducibility (Fig. 6d). This suggests that InrR is either stabilizing BisDC – as suggested, or stabilizing the mRNA and post-transcriptionally controlling ICE gene expression.*

*Action: We speculate on the role of BisDC and InrR in the discussion on p. 17, l. 1-10. No further changes.*

5. Are there similar DNA sequences in each promoter region that could represent potential binding sites for one or more regulators?

*Reply and action : We thank the reviewer for the suggestion and have made a promoter sequence comparison, which we have added to the manuscript as supplementary figure 3. There was indeed a common sequence motif in all promoter regions except PbisR, which perhaps reflects transcription factor binding sites. P. 7, l.24-26; p.8, l.23, p.15, l.4-6, p. 24, l.22-25.*

6. Fig4; Is there a mistake in 4d with PinR/Pint with PinR on the y-axis? all the other panels have Pint on the y-axis. Also, the reporter is indicated as Pint-eGFP (x axis) whereas all the others use Pint-mCHE (on the y-axis).

*Reply: We thank the reviewer for the comment. But no, there is no mistake. This was the only construct where the promoter reporter fusions were inverted.*

*Action: We have repeated the experiment with a strain that has Pint-mChe and PinR-eGFP, and replaced those data to avoid confusion. (Fig. 4c new panel)*

7. also fig4; It seems that the fluorescence from Pint-mCHE is different between several of the panels: ~150-200 in a; ~50-100 in c; ~400 in f, etc. What is the cause of these differences? Should they not all be similar?

*Reply: See our remark above to Reviewer 1. The differences here are real and this has puzzled us as well, even though the Pint-mCHE is in the same chromosomal location. Partly the effect comes from two camera systems have been used, as our data collection spans several years here. We further presume that this effect comes from transcriptional noise caused by low abundance transcription factors, that change binding when more target sequences become available.*

*Action: As suggested, we display these panels now as normalized fluorescence plots (normalized to the maximum observed fluorescence in the data series and subtracted from background fluorescence, p. 21, l.7-13 and legend to Figure 4). We discuss limitations of the quantile-quantile analysis on p.15, l.8-15.*

8. p10, line 14; regarding bisR expression in ~2x more cells than expression from other promoters; this could indicate additional regulators as suggested. It could also indicate threshold effects for BisR, as implied in the discussion.

*Reply: Indeed, another interesting point, which we were unable to solve so far. Previous stochastic modeling had suggested that even if BisR is bimodally expressed in a population, the level from the feedback loop is only halve (ref 26). Furthermore, even in absence of inrR, the expression of BisR is directly scalable to a proportional fluorescence reporter output from a promoter such as Pint (ref. 26). However, we find here that the alpA promoter is also controlled by InrR, so there may be additional feedback loops that we didn’t model.*

*Action: We revisited the discussion of the hierarchical network model in tc cells on p. 16 to better describe this. However, we don’t have any data to suggest that yet other regulatory factors should be involved – therefore, we chose not to speculate much more here and only update the regulatory model with the involvement of InrR.*

9. p7; regarding the RNA-seq data; My sense is that these data largely confirm previously published work from Northern blots and microarrays. Please indicate what is added here that was not previously published. Perhaps a better understanding of the location of the 5'-ends of the mRNA? These are consistent with the promoter activities measured with fusions. Also, please be careful not to imply that the RNA-seq data is measuring RNA synthesis, or even transcription start sites. The data identify the approximate location of 5'-ends and RNA abundance (not synthesis).

*Reply : The RNA-seq data were used for two purposes. First, to show transcription differences at higher resolution than previous Northern blots and micro-arrays and at different growth conditions (e.g., 3-CBA exponential and stationary phase, and succinate and 3-CBA stationary phases). Secondly, to show the effect of the mfsR and double mfsR/bisR deletion on ICE gene expression – to provide further evidence for our conclusion that the cascade of tciR-bisR-bisDC forms a concise regulon in tc cells.*

*We are aware that RNA-seq as performed here cannot show transcription start sites, although mapped transcript abundances and their directions are useful support.*

*Action: We removed Fig. 3 and combined it with Fig. S2 to form a single supplementary figure. The text was modified throughout to reflect the fact that we are looking here only at RNA abundances.*

10. The experiments seem to switch back and forth between P. knackmussii and P. putida, and at least one promoter appears to be active in P. putida and not P. knackmussii. Is there a reason for switching between the two organisms for different experiments?

*Reply : We apologize if this caused any confusion. We never managed to obtain ICE-free P. knackmussii* (which has two identical ICE copies)*; and, therefore, the effect of BisDC in isolation had to be conducted in P. putida. For unknown reasons, we did not manage to obtain a proper P. knackmussii clone with an ectopic promoter insertion of P-traI. Therefore, this promoter was the only one tested in P. putida with the ICE. ICE transfer rates and stationary phase induction are the same in P. putida as in P. knackmussii as we have shown on multiple occasions. Therefore, we believe that our conclusions on the ICE tc regulon are not falsified by the deployment of both strains.*

*Action: We explained this rationale in the introduction on p. 6, l.1-10, p.10, l. 3-5, and at further relevant places in the figure legends.*

11. I find the use of 'orthogonal' quite confusing and unnecessary. In some places, I'm not even sure what it means. For example; abstract, line 12; 'coordinately and orthogonally expressed'; orthogonal to what? not each other as they are 'coordinate'. There is differential expression relative to other ICEclc genes/promoters, and perhaps to many chromosomal genes, but perhaps not all.

*Reply : See our remarks above to reviewer 1. It was not our intention to use this term unnecessarily, but to find a term that would describe the transfer competence pathway regulation.*

*Action: We avoided the use of orthogonality in the title and the introduction, and changed the discussion of the regulatory hierarchy correspondingly (p. 16, l.14-p.17, l.10).*

12. Similarly, intro, line 11; 'maintain orthogonal components'; I think this is simply that MGEs have their own program(s) of gene expression. Different regulons within any bacterium have their own program of gene expression, and for a given regulon, there is some coordination and this is different from expression of other regulons. NO need to use 'orthogonal', which in my opinion complicates a very simple concept.

*Reply : See remarks above. We felt that the interesting message here was that the MGE has evolved a system to operate independently from the host in some cells, without affecting the other cells. In contrast to other regulons that have their own program of gene expression, the one from the ICE is additionally only active in a specific subpopulation of cells. This would imply some form of shielding or exclusion of promoter expression in non-tc cells, which we figured would be best covered with the term orthogonality.*

*Action: We removed orthogonality from the discussion on p. 16 l. 14-26; and changed the introduction on p. 4, l. 11-18 to explain the host-MGE network in more general terms.*

13. Abstract, line 6; change 'stands' to 'is a'; similarly, p4, line 17; 'stands model' should be 'stands as a model' or, more simply 'is a model'

*Reply : Thank you. Corrected.*

14. Table 1. regarding the proportion of stationary phase cells expressing reporters; clearly, MfsR is a repressor; but some promoters still only expressed in subpopulation in ∆mfsR cells, and in very different subpopulations. What does this mean?

*Reply : Thank you. The fact that we measure ‘subpopulations’ has to do with the experimental method. Strictly speaken, the qq-plot method is only accurate at very low subpopulation levels (~1-10%). At high levels, the ‘main’ population cannot be distinguished from the ‘sub’-population.*

*Action: We explained this in the text on p. 21, l. 3-5 and a footnote to Table 1.*

15. Table 1, footnote e; 'data reproduced from ref 58'; are the data taken from ref58 and shown here for comparison? or was the same experiment redone? I suspect that the data were taken from ref 58 (I did not check).

*Reply and action: The images were taken from ref58 and reprocessed for the data shown here. This was explained in the footnote to table 1.*

16. It would be very helpful to put Fig. 8 early, perhaps as Fig2, and use it to highlight what was known, and preview what is to come.

*Reply and action: Thank you for the suggestion. We moved Fig. 8 to Fig. 2 and hope it improved the comprehension of the work. See revised introduction text on p.5, l.14-26.*

Reviewer #3: This MS describes the transcriptional program of ICE activation for DNA transfer. It is a complex study building on previous work suggesting that ICE activation was limited to a small proportion of the bacterial population by a series of bistable switches. The authors use an impressive array of experimental approaches to try and identify the transcriptional cascade necessary for commitment to DNA transfer. Their most compelling data uses a set of fluorescent transcriptional reporters that are expressed from ICE promoters specifically activated in stationary phase. They further identify several promoters that are coordinately expressed in the same sub-population of cells suggesting a defined synchronized activation pathway for key conjugation genes.

*Reply : We thank the reviewer for the positive comments.*

The hypothesis makes sense – if cells are to conjugate then activation needs to be coordinated through a series of independent, parallel steps that ensure the cell is fully committed for trasnfer. The data in general are supportive of the model, but the individual experiments are not convincing and, thus, raise doubt about the conclusions drawn and the overall proposed mechanism of activation.

*Reply: We respectfully disagree to the reviewer that our individual experiments are not convincing to support the model. We provide several lines of evidence to be able to support our hypothesis (details commented on further below).*

Below I have focused on the figures as discussion points.
Figure 2 sets the stage for the reporter system by identifying 7 promoters that appear to be bimodal. The scales in 2b vary dramatically from 100-1000, especially for the P.putida data set and 50240. This particular gene reporter is an outlier in many subsequent experiments, and it is not clear to this reviewer if these data can be properly compared with the other promoters in a different strain background. Why are the fluorescent levels so different? They do not correlate with promoter strength.

*Reply: We thank the reviewer for this observation. Indeed, these promoters appear to be very different in strength. This can be appreciated from the RNAseq data as well. However, there is no ground that genes being part of a regulon can not be individually expressed to different levels, because this is determined by the local promoter context, and further post-transcriptional or post-translational processes.*

*We do acknowledge that, when testing ectopic promoter expression, we forcibly use a fragment that we hope contained all the necessary elements, but that is taken out of context. Therefore, this can have consequences both at the high and the low scale. Furthermore, as the scatter plots attest – having different combinations of paired promoters can also influence reporter output.*

*Finally, as explained to both other reviewers, two camera systems were used as our data collection spans several years here.*

*For these reasons, we don’t specifically interpret the promoter strengths, but rather the subpopulation sizes of deduced tc cells, which show the consistency of the data.*

*Action: We rephrased the text on p. 7, l.1-2 to clarify this. Promoter variation was commented on p.7, l.24-26. We comment on the limitations of qq-plotting on p.15, l. 8-15. Copy interference was discussed on p.16 l.22-26*.

Fig 3 presents RNA-seq data but the interpretation of these is not clear in the text. The data are poorly presented. Transcript reads are not assigned to a strand, so it is impossible to determine directionality. The read coverage axes are very different and, in many cases, not adjusted to the reads of the relevant gene. E.g., P50240 scale at 0-800 is set to a gene in the opposite direction, when 50240 is expressed at very low levels, similar to int, while the Pint scale is appropriately set to 100 reads. Many of the views are not optimized for read depth. The 5’ of RNA-seq should identify the transcriptional start site. In many cases the “promoter arrow” does not coincide with the 5’ end of reads. Why? Eg., PinR, P81655, P50240, PalpA. Is the precise TSS really known or are these best guesses? The precise identification of each TSS would also provide confidence that the correct a-r promoter segments have been cloned (Fig. 1b, P7 25- and P13 21-23).

*Reply: We thank the reviewer for the remark. Indeed, this presentation does not allow directionality. In fact, the method itself as we implemented does not allow proper direction estimation (although we can see the direction of individually mapped reads from placed reads in BamView). We adjusted the read coverage to be consistent between regions, as much as possible (for example, most panels go from 0-600); and we aimed to show the difference here between exponential and stationary phase transcript abundances on two different carbon substrates (which induce or not tc cell formation).*

*As Reviewer 2 pointed out, RNA-seq cannot be used to determine 5’ start sites, but is only indicative. Furthermore, the RNA-seq interpretation is difficult, because we assume from our hypothesis that the interesting ‘signal’ should come from 2-5% of tc cells in the total population. Therefore, we use the read abundance plots as support for the measured reporter activity from cloned fragments, and under different growth conditions – and we place the promoter arrows as indications for them being part of the tc cell regulon. Unfortunately, 5’RACE was too noisy to precisely map promoter starts in tc cells. We hope this can be improved in future work with single cell transcriptomics but feel this is outside the current scope of what we can achieve.*

*Action: We rephrased text and interpretations from RNAseq data on p. 8, l.11 – p.9, l. 16 and moved Fig. 3 to supplementary figure 2, where it is combined with the RNAseq data from succinate-grown cells.*

RNA seq cannot distinguish between new starts and readthrough making assigning promoter activity very difficult (e.g., 58432, see P7 line 20-21). Similarly, readthrough in RNA seq does not allow one to rule out promoter activity in UR62755,66202 etc. (P8 1-3). As these promoters are activated only in 2-5% of cells it is impossible to interpret reads in tc cells vs the overall population. The authors mention this in passing, but fail to use this knowledge in their data interpretation. The RNA seq data show that essentially all of these regions are transcribed preferentially during stationary phase, regardless of whether the region contains a putative tc promoter (P7, 14-17, p8, 17-19).

*Reply: We thank the reviewer for this observation. As we reply above, it is currently not possible to see reads coming only from tc cells. We have (unsuccessfully) tried this by using FACS-sorted tc cells but this sorting was insufficiently precise. We hope to improve this in future research by single tc cell transcriptomics.*

*Importantly, we do not rely on a single method to draw conclusions, and these limitations are the reason why we use a combination of single cell single-copy fluorescent reporter gene studies, RNAseq and mutant analysis to interpret the nature of the various cloned fragments and their assumed promoters.*

*Action: We revisited the text passages on p. 8 and p. 9 and modified them where necessary.*

Fig 4. The scales for Fig4 are highly variable, and yet all are compared to Pint – the internal control. Mcherry Int-positive cells increase from a cut-off of 105 (c) to 180 (a) and 500 (f). Why?
Label on (d) is switched compared with axes.

*Reply: We apologize for any confusion. As mentioned above, the label was not switched but this was a strain carrying a Pint-eGFP/PinR-mCherry fusion. This was repeated with a strain carrying the reverse (new Fig 4c panel). Cut-offs are set by qq-plotting, which we explain more clearly in the revised materials section.*

*Fluorescence intensities are different because of (i) differences in inherent promoter strength, and (ii) transcriptional noise arising from having different copies of paired promoter combinations.*

*Action: We now display Fig. 4 as background-corrected, maximum-normalized fluorescence intensities.*

The data for 4a-e are compelling, but f-g are less clear and suggest f-g are not coordinately expressed with Pint. Yet P9;10-11 states that only bisR is not coordinately expressed. Authors suggest that 58432 is not clear because of it having a weaker promoter. But its promoter is as strong as 81655 and 88440 (Fig 2d), so this argument appears flawed. 50240 is an outlier again as it is only expressed in Pput. Despite having made the argument for inclusion of 50240 and 58432 as part of the tc regulon (p9 top paragraph), the authors exclude these two promoters from further analysis because of their poor correlation (P9 bottom paragraph). This is appropriate given their poorer correlations, but the authors still include these two genes in their discussion and model regulon (Fig 8).

*Reply : We thank the reviewer for bringing up this discussion. The reasons for some promoter being stronger and others weaker expressed, and for additive effects are not clear. We suspect this is due to stochastic variation and copy number titer effects (see discussion above). For this reason, we try to base our conclusions on different lines of evidence: (i) promoter activity in isolation, (ii) correlation to expression of Pint, (iii) RNAseq, (iv) heterologous expression in P. putida and (v) dependencies on the major transcription factors coordinating tc development: MfsR, BisDC and InrR. Finally, the local sequence context of the promoter motifs also determines its ‘strength’. By including a promoter search, we find a common motif that could be a transcription factor binding site. We believe that this is a good as we can get it, and justifies that even if one out of five criteria is weaker, it still counts as a promoter being part of the proposed regulon.*

*We repeated time-lapse experiments with all the promoter combinations, but also here there is a clear influence on expressed reporter fluorescence intensities from the combination itself (see new Figure 5a)*

*The choice of testing P50240 in P. putida and not P. knackmussii was purely technical. For unknown reasons (as we mentioned), we did not manage to obtain proper constructs for this promoter in P. knackmussii, but only P. putida with the ICE. However, because the ICE can operate in both hosts and transfers with similar rates from both donors, we consider it justified to conclude on the restriction of this promoter being expressed in tc cells, even though it is in a different host.*

*Action: We repeated time-lapse experiments and rephrased the interpretations to accommodate the reviewer’s comments. This was presented in a completely revised Figure 5, additional parts to the methods sections on p. 21/22 and rewriting the results section of Figure 5 starting on p.10, l. 10. We acknowledge throughout the text that P58432 results remain inconclusive, because not all criteria for promoter activity are fulfilled (e.g., p.9, l.13-16).*

Fig 5 and S3. Data for 67231 are not included in Fig 5 or fig S3 despite it clearly belonging to the tc regulon. These are complex figures that need better explanation and presentation. It is clear that bisR is different from the others. What is not clear, especially in Fig 5a, is the co-expression of fluorescence in both cells because the figs are too noisy. S3 does this a bit better as the traces are labelled. Perhaps a more convincing approach would be to provide a few graphs with no background and showing just a few cells with onset for both reporters?

*Reply and action: We thank the reviewer for this suggestion. We conducted time-lapse experiments for the remaining constructs, re-analysed more cell data for all, and reworked this figure according to the suggestions by limiting and color-labeling the number of traces. We also include a new method to analyse transcription start timing. See our reply to the previous remark.*

5d/e need more explanation as I came to a very different conclusion to the reviewers on P10;3-15, when they suggest that the timing onset for all was well correlated except for BisR. What seems clear to me is that Pint and P81655 exhibit early expression in both 5a and S3a with expression onset in the 5-10 hr window. By contrast for the other three constructs, Fig S3 indicates their first fluorescent response is in the 1000-1500 min window (16 hrs). This is especially clear for S3c/d, in which there are no signals in the first 1000 mins. This observation is further supported in 5de, when comparing 81655 and int to other promoters; their first brown squares (d; left graph) co-express at 10 hrs vs 20 hrs for all others. The histograms in (e) follow the same pattern. The interpretation of this figure is key to the overall model (fig 8, P12,9-13 and discussion), which posits that all of these promoters are coordinately induced at the final stage of the regulon. My interpretation of the data suggests that Pint and 81655 are induced earlier.

*Reply and action: We thank the reviewer for the suggestion and interpretation. We have included more cell data from time-lapse experiments and designed a new procedure to automatically distinguish tc cell traces and to derive expression starts. We are confident that this helped to improve our interpretation here. See text passages and new figure 5 referred to above.*

Additional issues with these two figures
5c, could the authors confirm the PalpA line of correlation is correct?
S3 needs correcting. Time is in mins not hours. The scales vary widely, even for mcherry. Why?
Some axes start at 0 others 500 min.
S3d/e traces are not labelled or colored to distinguish cells

*Reply: These figures are reworked to correct these details. We decided to now show relevant paired traces for all promoter pairs and include a new way to determine the starting time of promoters in tc cell populations (Fig. 5b and new supplementary figure S4).*

Fig 6a the labels are too small. It is nice to show the entire ICE, but the relevant regions should be expanded so gene names are eligible, especially those discussed in the document.
6b please explain the bisR fold change compared to wt, when the bisR gene is deleted.
6d Please explain why there are two copies of InrR? P12,3-6. The second copy is not shown in Fig 1.

*Reply: There are two copies of inrR because there are two identical ICE copies in the genome of P. knackmussii. We explain this in the text on page 6, l. 2; p. 4, l. 25, and p. 13, l.11-13.*

*Action: We improved labels in Fig. 6a and corrected the bisR calculation.*

Fig 7 lacks detail and logic. The goal is to understand the function of the tc regulon genes (P12;15-21). These experiments do not address that question in a meaningful way. What is surprising is that substantial deletions of multiple regulon genes has no impact on conjugation. Why were these regions deleted in particular? Why were such large deletions created, rather than promoter deletions? Did the deletions include the promoters or just the genes? virD4 is essential for transfer, so its deletion along with other genes provides no insight on the function of the other genes; that deletion was always going to be transfer defective.

*Reply: We thank the reviewer for this comment. The goal here was not to create individual promoter deletions and study their effects, but to get a broad idea of whether conserved gene clusters are important for ICE transfer. Making individual gene deletions would be beyond the scope and breadth of the current work.*

*Action: We rephrased the text and its justification on p. 6, l. 10-12, p. 13, l. 22-23 and p.16, l.1-12.*

General comments
The word usage and syntax need addressing. I understand that English is not the native language; but the text would benefit from some editing.

*True! We combed again through the final revised text to improve it as much as possible.*

This is a very complicated series of experiments involving many different genes and promoters, which made the work extremely difficult to follow. There are a-r gene segments, containing different promoters, with confusing 5-digit labels and names in small subscript . Fig 2 continues to use both the letter and gene assignment, but I don’t think this helps without having fig 1 on display. The lineups of the individual charts are different in each fig 2-5, making it difficult to follow, especially when the promoter labels are in subscript. I am not sure there is a simple solution, but readability would be dramatically enhanced by dropping the multi-digit labels and using a standard flow for each figure.

*Reply and action : We thank for this comment and apologize that the initial gene nomenclature was not ideal from the perspective of presentation (but we have to keep this to avoid confusion with previous ICEclc work). We did our best to revisit the a-r gene segments and make sure they were used wherever relevant, and maintained the order ot promoter presentations the same in all figures. On the basis of suggestions by reviewer 2 we now present the model earlier in the text, hoping this helps interpretation.*
