# Supplementary material for: A bistable prokaryotic differentiation system underlying development of conjugative transfer competence
Source: PLoS Genet. 2022 Jun 28;18(6):e1010286. doi: 10.1371/journal.pgen.1010286 (PMC9286271; doi:10.1371/journal.pgen.1010286)
Supplement: Attachment — Submitted filename: Reply_reviewer3.docx [file pgen.1010286.s015.docx]

**Reply to the comments of reviewer 3:**

1. Fig 7 shows the impact of large deletions of three regions. I queried this before and the authors have not addressed the issue. Fig and text are essentially the same and are misleading (p13 and 16, 1-12).

*Reply : We appreciate the concerns of this reviewer but do not see how our text is misleading. We did not change this part of the text, because we could not add more information and according to us, all information is correctly presented and transparent. To create additional gene deletions was, in our reasoning, out of the scope for this manuscript, where we essentially focus on characterizing all bistable promoters, but not characterizing all ICE gene functions.*

One region is required for transfer. But it includes the known transfer gene virB4, so the role of the other ~10 genes cannot be interpreted. On P16, the authors ignore this fact and suggest that P81655 transcribes the second half of the operon.

*All the evidence presented in this work indicates that the promoter upstream of orf81655 is the only promoter that drives expression of all genes from orf81655 until orf68241 after virD4. We do not interpret the role of the other 10 genes. We tested an intergenic fragment (q) upstream of orf73676, where previous transcript mapping had shown a breakpoint. However, this showed no promoter activity (Fig. 3). Together with the previously established Northern mappings, this indicates that P-orf81655 is a single promoter. Transcriptomics (Fig. 6 and Fig. S) is in line with this.*

There is no evidence for this and the authors cannot rule out there are downstream internal promoters driving virB4 expression. The other region deleted is also not required for transfer (fig 7).

*With all respect, but we don't understand what this reviewer means with 'there is no evidence for this'. Heterologous promoter expression (done here), transcriptomics (done here), Northern mapping (done in Gaillard 2010) all indicate that there is no other internal promoter. It is thus extremely unlikely that there would be an additional promoter, and given the principle of Occam's razor, we should strive for the simplest coherent explanation. However, as we acknowledge already on P. 8, l.18-20, and on p. 15, l. 10 there is always a possibility that our tested fragments did not completely cover everything. We therefore feel there is no need to add further ‘disclaimers’ here.*

Yet they continue to speculate that these large operons are “functionally important for ICE regulation and/or transfer???”(P16 10-12).

*Reply : As we mention on p. 16, l. 6-9: 'By contrast, the role of the genes...is not clear, since their deletion...'. We acknowledge that their deletion had no effect on transfer. Our only argument (l. 10-12) is their wide gene and synteny conservation (Fig. S6), suggesting that they have a function for the ICE. We believe this is not misleading, but a justified discussion and suggestion.*

And, if they are really part of the regulon that primes cells for transfer, how can their deletion have no impact on transfer? There is no interpretation of how these surprising results support their model.

*Reply : With all due respect; if we knew this, we would have written it differently. One suggestion we have ourselves is that transfer was only tested between isogenic strains (as mentioned on p. 16, l. 8). It might be that these orfs have some role in mating between unrelated strains. Again, we believe we explained this clearly - results are transparent, conclusions seem justified; what is misleading here?*

*Action : we have changed (underlining) the sentence on p. 16, l. 5-7, to:*

However, since many of them are conserved among ICEs of the same family in different hosts both by individual sequence as well as in gene synteny (S6 Fig), it is likely that they have functional importance for some aspect of ICE maintenance, regulation and/or transfer.

2. They identify a potential consensus sequence (S3) but we are not told where the motif maps relative to transcription and/or the gene.

*Reply and action: We thank the reviewer for this comment. The distance to the start of the gene was presented in Fig. S3. We added the distance to the mapped transcription start in the inrR-promoter that had been previously determined (Minoia et al., 2008). Results from 5’RACE for other promoters have not been reliably determined, therefore we prefer not to indicate them.*

As presented this is not convincing, especially as the numbers indicate very different distances to the downstream gene(?).

*Reply and action : We appreciate the comment and we acknowledge that we do not have better information here. Bioinformatic motif searches can only tell so much. There is no genetic rule that says that transcription factor binding sites should be at the same distance from the start codon, or even with respect to the RNA polymerase binding positions.*

*We added the calculated p-values of the MEME program to Figure S3 and added another disclaimer to the text on p.14/15:*

The common detected promoter sequence motif corroborates coordinated transcription regulation, although further studies are needed to confirm actual BisDC binding sites.

3. Although writing is improved the text is still not clear and word usage increases the difficulty of interpretation. Even the abstract has missing words and poor sentence construction. More care is needed in the text and figs to avoid making me concerned about other points I have missed and their overall rigor – also suggested by their data interpretation. Eg lack of uniformity in text and figures for TraI and 50240, which I realized eventually are the same gene! Same for Int and IntB13, and InrR and InR. Fig S1 promoter fragments are all in the opposite orientation to Fig 1.

*Reply : We thank the reviewer for these details. We did not spot missing words in the abstract and we do not understand what is implied with ‘..missed…overall rigor – also suggested by their data interpretation’. Our data are transparently presented with all their background and numerical values; therefore there is no reason to imply ‘sloppiness’.*

*Despite having screened multiple times, apparently two of these notation errors have escaped us. The double notation of orf50240/traI was already presented in Figure 1.*

*Of note: the different notation for the promoter and the gene intB13 (P-int) is deliberate and has been in use by us since 2007 (!); same for PinR and the gene inrR (we use the capital 'R' here to avoid confusion with Pint; since a small 'r' and 't' look very much alike).*

*Action: Fig S1 fragments were mirrored.*
